# Supplementary figures and images for: Body muscle gain and markers of cardiovascular disease susceptibility in young adulthood: A cohort study
Source: PLoS Med. 2021 Sep 9;18(9):e1003751. doi: 10.1371/journal.pmed.1003751 (PMC8428664; doi:10.1371/journal.pmed.1003751)

**S1 Fig** Sex-specific distributions of lean and fat mass indices (kg/m<sup>2</sup>) at age 10y

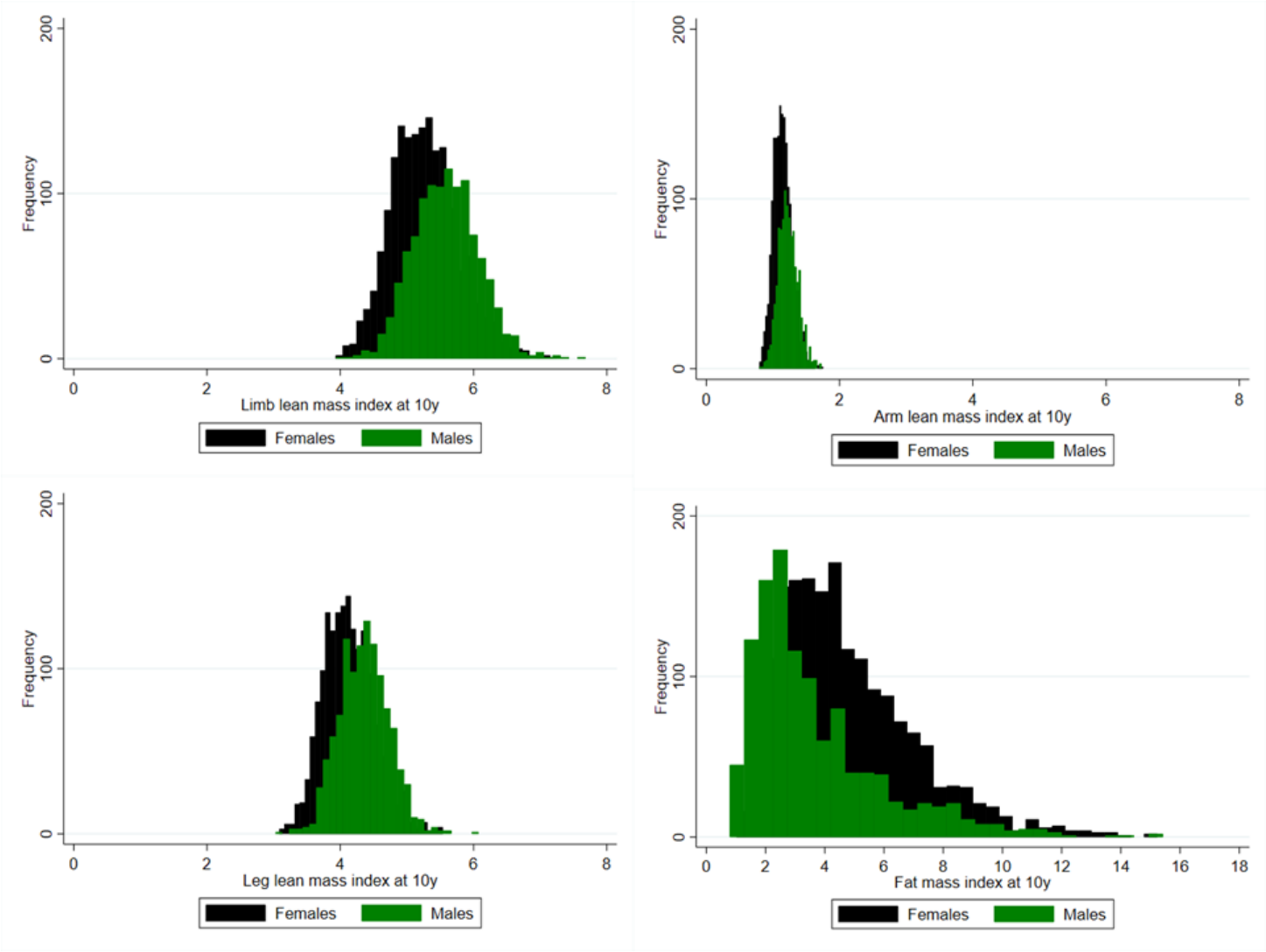

Supplement: S1 Fig — (PDF) [file pmed.1003751.s002.pdf]

**S2 Fig** Sex-specific distributions of lean and fat mass indices (kg/m<sup>2</sup>) at age 13y

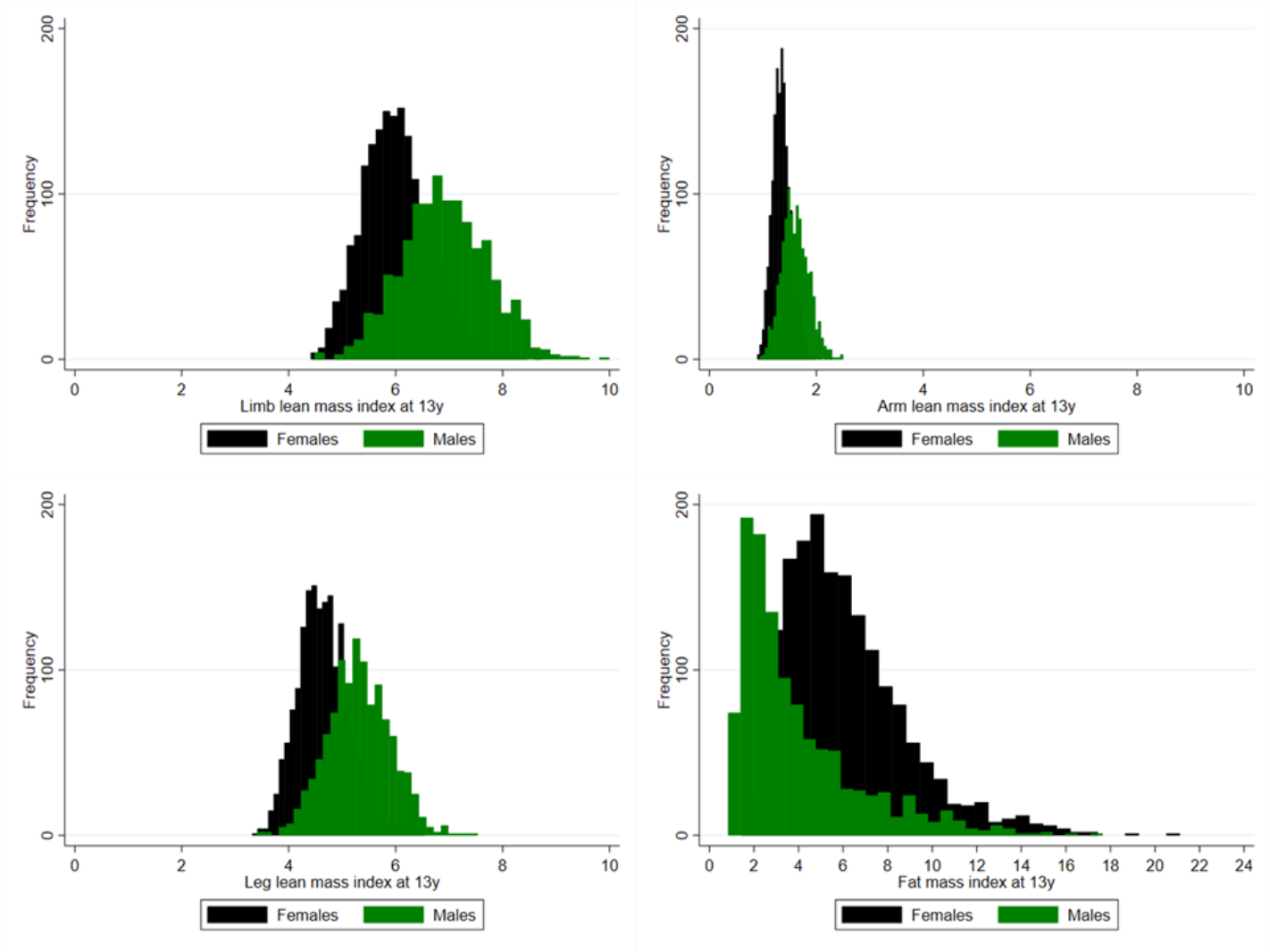

Supplement: S2 Fig — (PDF) [file pmed.1003751.s003.pdf]

**S3 Fig** Sex-specific distributions of lean and fat mass indices (kg/m<sup>2</sup>) at age 18y

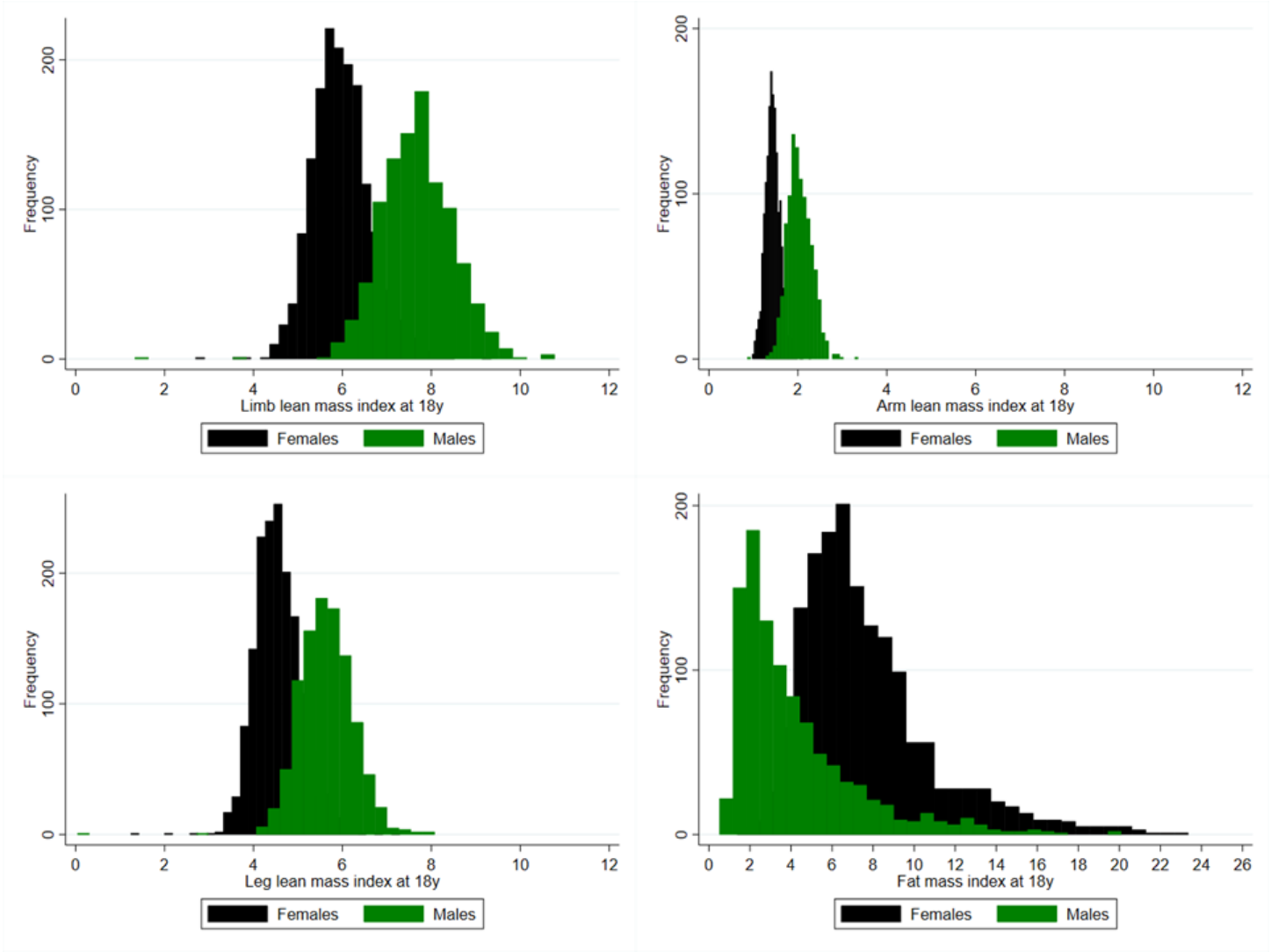

Supplement: S3 Fig — (PDF) [file pmed.1003751.s004.pdf]

**S4 Fig** Sex-specific distributions of lean and fat mass indices (kg/m<sup>2</sup>) at age 25y

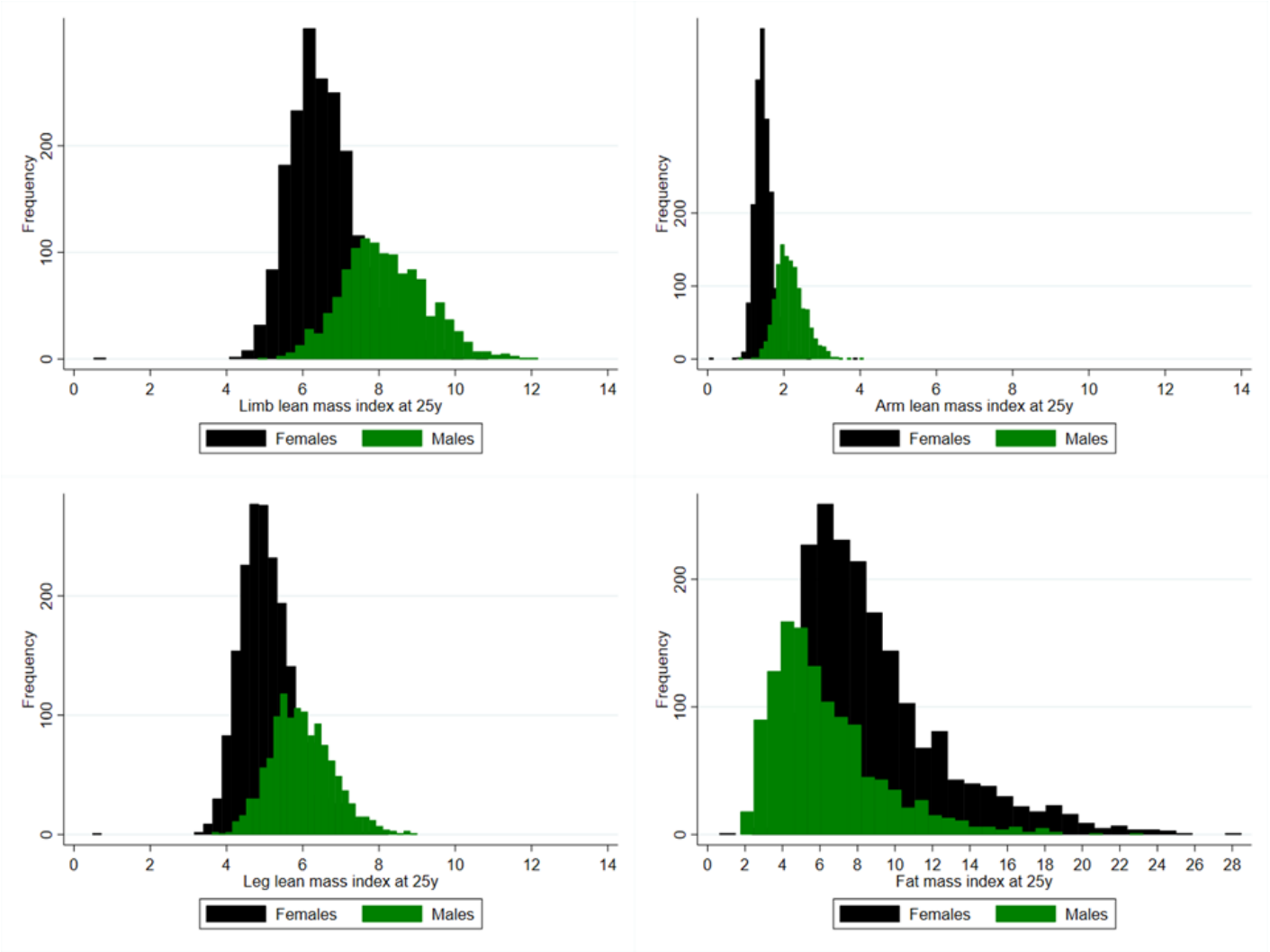

Supplement: S4 Fig — (PDF) [file pmed.1003751.s005.pdf]
