# Supplementary material for: Body muscle gain and markers of cardiovascular disease susceptibility in young adulthood: A cohort study
Source: PLoS Med. 2021 Sep 9;18(9):e1003751. doi: 10.1371/journal.pmed.1003751 (PMC8428664; doi:10.1371/journal.pmed.1003751)
Supplement: S5 Fig — Units of maximum grip are kilograms at age 12 y and pounds per square inch at age 25 y. Units of relative grip are kilograms/weight in kilograms at age 12 y and pounds per square inch/weight in kilograms at age 25 y. (PDF) [file pmed.1003751.s006.pdf]

S5 Fig Sex-specific distributions of maximum and relative grip strength in childhood and young adulthood

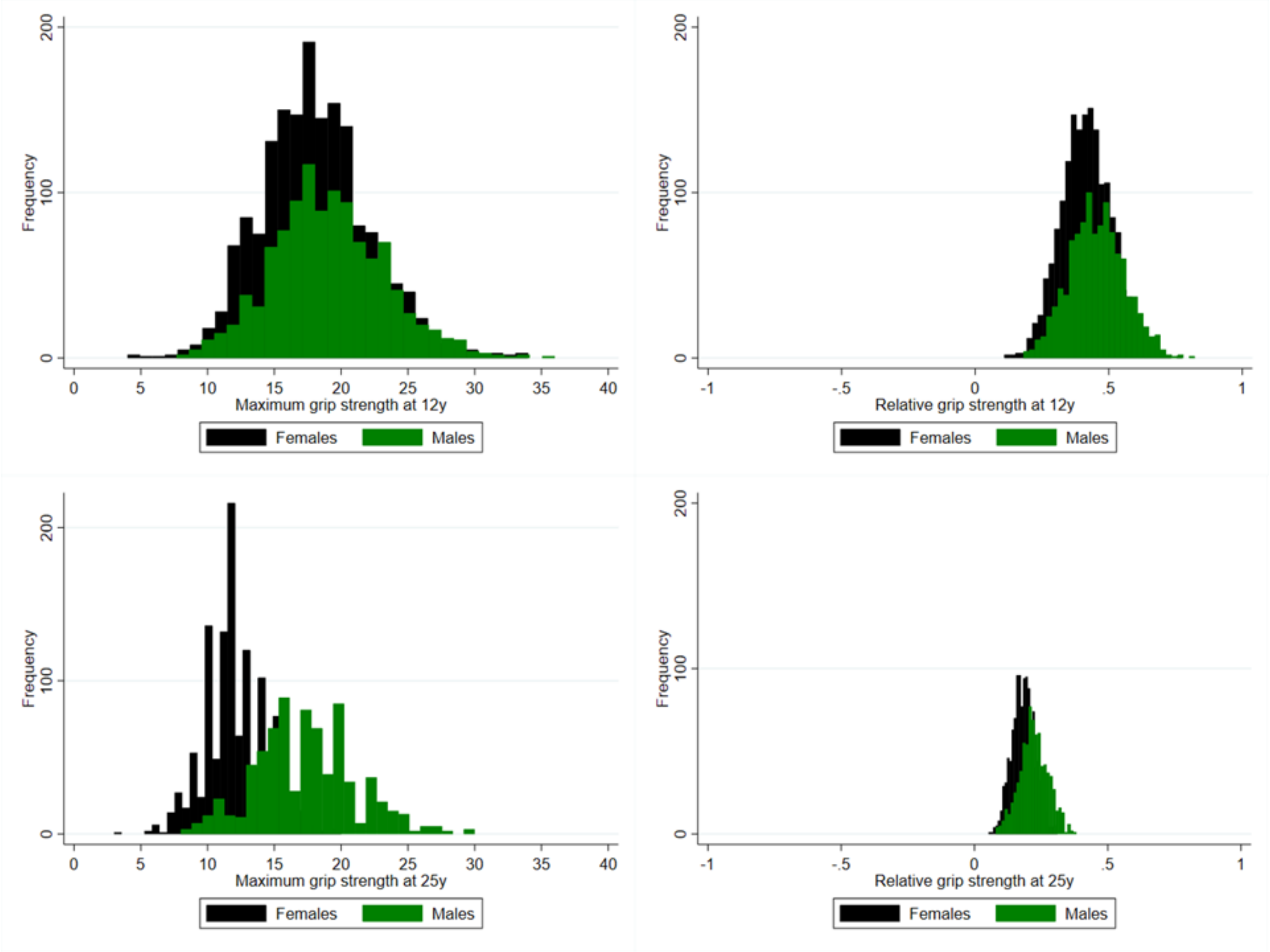

Units of maximum grip are kg at age 12y and lb/in^2 at age 25y. Units of relative grip are kg / weight in kg at age 12y and lb/in^2 / weight in kg at age 25y.
