# Supplementary material for: Body muscle gain and markers of cardiovascular disease susceptibility in young adulthood: A cohort study
Source: PLoS Med. 2021 Sep 9;18(9):e1003751. doi: 10.1371/journal.pmed.1003751 (PMC8428664; doi:10.1371/journal.pmed.1003751)
Supplement: S8 Fig — Change values are based on difference scores (18-y value minus 13-y value), in original units (kg/m2). (PDF) [file pmed.1003751.s009.pdf]

S8 Fig Sex-specific changes in lean and fat mass indices in adolescence

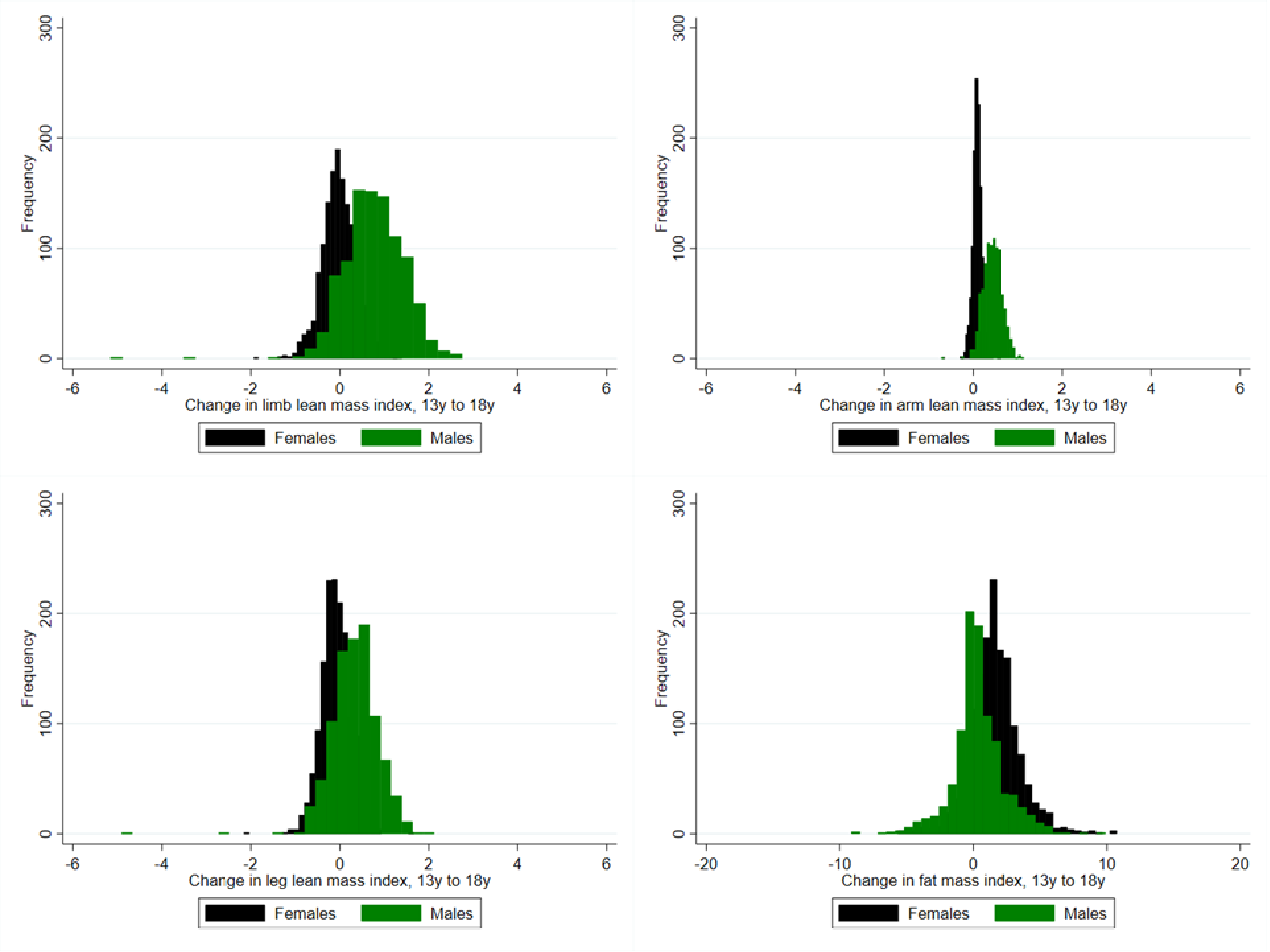

Change values are based on difference scores (18y value minus 13y value), in original units (kg/m^2).
