# Supplementary material for: Body muscle gain and markers of cardiovascular disease susceptibility in young adulthood: A cohort study
Source: PLoS Med. 2021 Sep 9;18(9):e1003751. doi: 10.1371/journal.pmed.1003751 (PMC8428664; doi:10.1371/journal.pmed.1003751)
Supplement: S9 Fig — Change values are based on difference scores (25-y value minus 18-y value), in original units (kg/m2). (PDF) [file pmed.1003751.s010.pdf]

S9 Fig Sex-specific changes in lean and fat mass indices in young adulthood

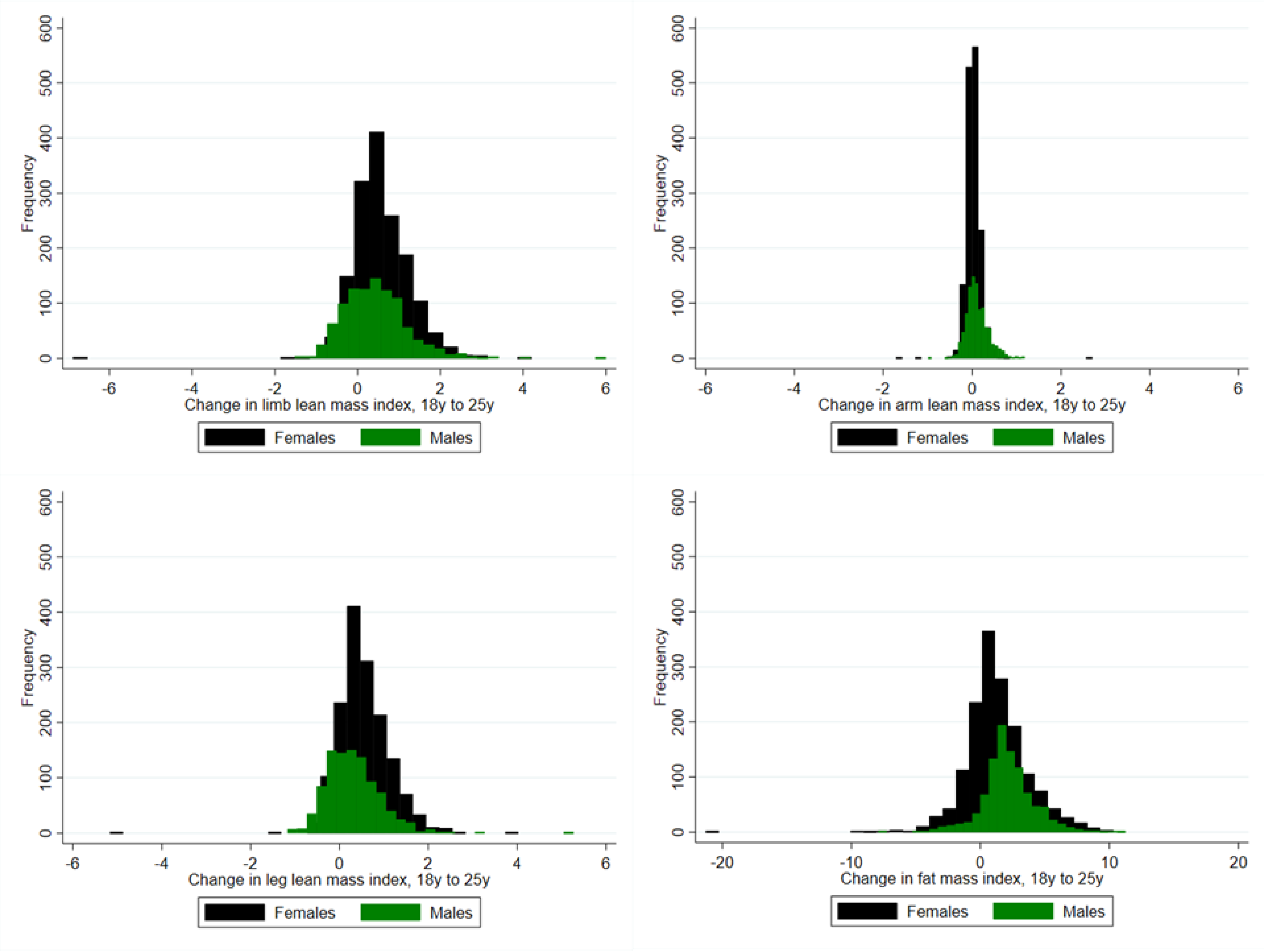

Change values are based on difference scores (25y value minus 18y value), in original units (kg/m^2).
