# Supplementary material for: Body muscle gain and markers of cardiovascular disease susceptibility in young adulthood: A cohort study
Source: PLoS Med. 2021 Sep 9;18(9):e1003751. doi: 10.1371/journal.pmed.1003751 (PMC8428664; doi:10.1371/journal.pmed.1003751)
Supplement: S10 Fig — Change values are based on difference in SD units (25-y z-score value minus 12-y z-score value), given the different original measurement units between occasions. (PDF) [file pmed.1003751.s011.pdf]

S10 Fig Sex-specific changes in handgrip strength from childhood to young adulthood

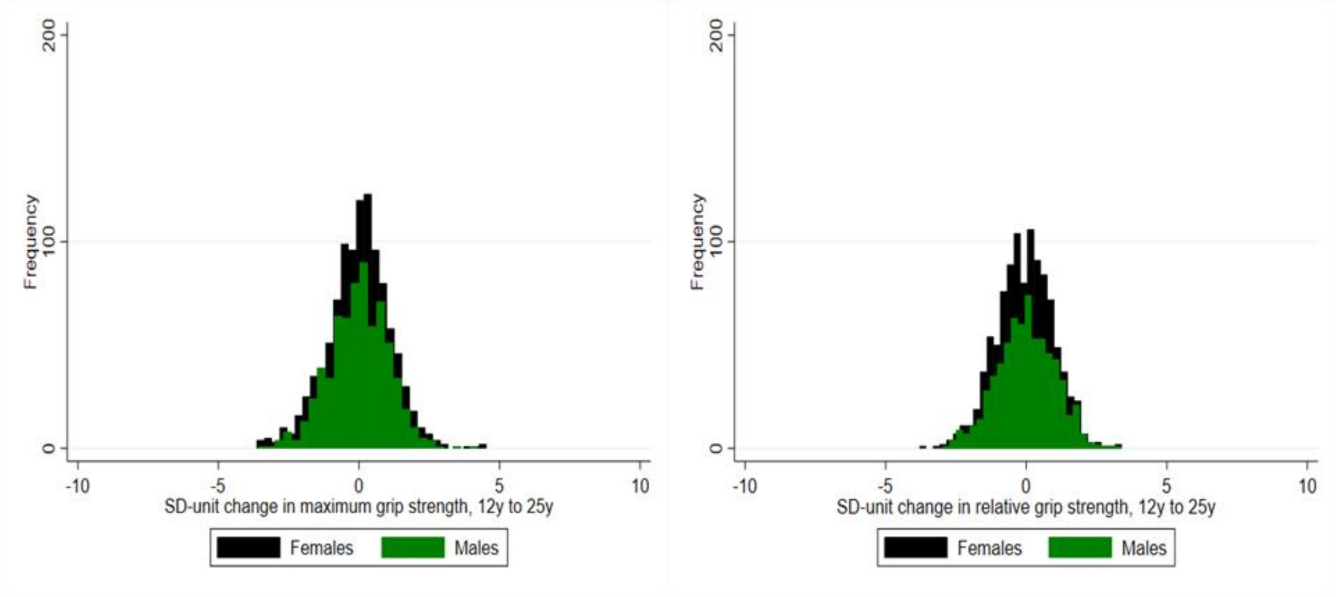

Change values are based on difference in SD units (25y z-score value minus 12y z-score value), given different original measurement units between occasions.
