# Supplementary material for: Body muscle gain and markers of cardiovascular disease susceptibility in young adulthood: A cohort study
Source: PLoS Med. 2021 Sep 9;18(9):e1003751. doi: 10.1371/journal.pmed.1003751 (PMC8428664; doi:10.1371/journal.pmed.1003751)
Supplement: S1 Study Plan — (PDF) [file pmed.1003751.s012.pdf]

Submission information

Impact of leanness on type 2 diabetes liability (B3277)

Submission date/time: 20/03/2019 04:14:57

Principal Applicant Details

Principal Applicant Title: Dr

Your name: Joshua Bell

Position Held (note: If you are a student, the proposal should be submitted by your supervisor with you listed as a co-applicant): Elizabeth Blackwell Institute (EBI) Early Career Fellow

Is this a student project?: No

What type of student project is this?:

Please specify what type of student project is this...:

Affiliation: MRC IEU, University of Bristol

Are you a 'direct access' user of ALSPAC data, or a member of the Integrative Epidemiology Unit (IEU)?: Yes

Address line 1: Oakfield House

Address line 2:

City: Bristol

Country: United Kingdom

Postcode: BS8 2BN

Email (Please use your institutional email address): j.bell@bristol.ac.uk

Telephone: +44 (0)11 7331 0085

Co-applicant details

How many co-applicants?: 3

Co-applicant 1

Name and title: Prof George Davey Smith
Position Held and Affiliation: Professor of Clinical Epidemiology & Director, MRC IEU
Email: kz.davey-smith@bristol.ac.uk
Role in Project: Supervisor/Line manager

Co-applicant 2

Name and title: Dr Kaitlin Wade
Position Held and Affiliation: EBI Early Career Fellow
Email: kaitlin.wade@bristol.ac.uk
Role in Project: Collaborator

Co-applicant 3

Name and title: Prof Nicholas Timpson
Position Held and Affiliation: Professor of Genetic Epidemiology & ALSPAC PI
Email: n.j.timpson@bristol.ac.uk
Role in Project: Collaborator

Co-applicant 4

Co-applicant 5

Co-applicant 6

Co-applicant 7

Co-applicant 8

Co-applicant 9

Co-applicant 10

PROJECT DETAILS

Title of project: Impact of leanness on type 2 diabetes liability

Proposed Project Start Date: 2019-04-01

Proposed Project Finish Date: 2021-04-01

Is your project currently funded?: Yes

Current project funding: Elizabeth Blackwell Institute/Wellcome Trust, University of Bristol

Are you seeking funding for this project?: No

----------------Potential funding fields----------------
Will your project be a cross-cohort project or part of a consortium?:

Please provide a description of how ALSPAC data will be combined with other datasets, how it will be managed and how many researchers are likely to access it.:

Have you previously had a project with us?: Yes

Data buddy name (if one has been assigned to you):

Where did you find out about ALSPAC?:

Please specify where you heard about ALSPAC?:

Research dissemination: Peer review journal article, Conference, Report

Other plans for disseminating research findings: Preliminary results will be discussed as part of fellowship applications

----------------Invoice details----------------
Name of legal signatory for your institution (e.g. representative from contracts department):

Link to the information security policy for your institution:

Description of Project

Project summary for laypersons: We know that obesity (high body fatness) is a major risk factor for type 2 diabetes. We also know that obesity is very difficult to reverse, and that we need to find ways of preventing type 2 diabetes when fat loss is not feasible. Muscle tissue – the other major body compartment – is likely beneficial for health, but little is known about which aspects of muscle (e.g. whether volume or strength) matter most for the earliest stages of type 2 diabetes,

and how these benefits compare with the harms of fat. This project aims to use repeated measures data from ALSPAC offspring and parents to determine which aspects of muscle – whether higher volume based on body scanning, higher strength based on hand grip tests, or higher quality based on a combination of strength and volume – has stronger effects on an extensive set of detailed metabolic traits related to type 2 diabetes susceptibility. It also aims to determine how benefits of muscle compare with harms of fat, and whether muscle interacts with fat in relation to early stages of disease. Altogether, these results should help us to better understand which aspects of body composition are most important to target with limited public resources in order to prevent type 2 diabetes, and whether boosting muscle would help prevent type 2 diabetes when fat loss is not feasible.

Aim(s) and objective(s): Aims

1. To compare the known harms of body fatness with the potential benefits of body leanness for type 2 diabetes liability in early life

2. To determine which aspects of body leanness – total and regional muscle mass, muscle strength, or muscle quality – most strongly influence type 2 diabetes liability in early life

3. To determine whether aspects of body leanness interact with body fatness in relation to type 2 diabetes liability in early life

4. To determine whether any benefits of leanness for type 2 diabetes liability seen in early life are replicated and more pronounced in later life

Objectives

1. To examine the pattern and magnitude of associations of higher total and regional lean mass estimated by dual-energy X-ray absorptiometry (DXA) body scans with metabolic trait levels estimated by targeted nuclear magnetic resonance (NMR) metabolomics and standard clinical assays among ALSPAC offspring

2. To examine the pattern and magnitude of associations of higher muscle strength estimated by hand grip strength with metabolic traits from NMR and clinical assays among ALSPAC offspring

3. To examine the pattern of magnitude of associations of higher muscle quality estimated by hand grip strength as a function of total and regional lean mass with metabolic traits from NMR and clinical assays among ALSPAC offspring

4. To examine evidence for statistical interaction between aspects of body leanness with body fatness in relation to metabolic traits from NMR and clinical assays among ALSPAC offspring

5. To repeat above objectives among ALSPAC parents to compare patterns and

magnitudes of trait results at older ages and examine risk of type 2 diabetes onset/diagnosis

Methods (including an overview of statistical methods): Data for the first set of analyses will come primarily from ALSPAC offspring participants who have measures of socio-demographic and health behavioural characteristics in addition to 6 measures of DXA body scans (at approximately age 10y, 12y, 13y, 15y, 18y, 24y), 3 measures of hand grip strength (at approximately age 12y, 18y, 24y), 4 measures of NMR metabolomics (at approximately age 8y, 15y, 18y, 24y), and 4 measures of insulin and C-reactive protein (at approximately age 10y, 15y, 18y, 24y).

The main exposures of interest are total lean mass (whole body DXA), regional lean mass (trunk, arms, and legs separately from DXA), hand grip strength, and 'muscle quality' estimated as hand grip strength as a function of total and regional lean mass.

Main outcomes will be circulating metabolic traits from NMR metabolomics (including various lipids in lipoprotein subclasses, fatty and amino acids, and inflammatory glycoprotein acetyls), plus circulating fasting insulin and C-reactive protein.

Main confounders of interest are sex, age, ethnicity, maternal education, height, total body mass, total and regional fat mass from DXA, puberty timing (estimated by age at peak height velocity, already derived), and post-pubertal smoking, alcohol consumption, and physical activity.

Linear regression models will be used to examine cross-sectional and prospective associations of each aspect of leanness (in standard deviation, SD, units) with metabolic trait outcomes (in SD units), adjusting for mentioned confounders at respective time points. Statistical interaction between different leanness measures and DXA fat measures will be examined using product terms within regression models.

Results will be compared with those produced previously for fat indexes in relation to these same metabolic outcome traits using data from ALSPAC offspring (e.g. Bell et al. Associations of Body Mass and Fat Indexes with Cardiometabolic Traits. J Am Coll Cardiol. 2018. 72. (24.)) to allow results to be interpreted and prioritised within a wider context.

Data for the second set of analyses will come from ALSPAC parent participants (mothers and fathers) who have measures of socio-demographic and health behavioural characteristics in addition to repeated measures of DXA-based total and regional lean mass (among mothers at FOM1, FOM2, FOM3, FOM4 and among fathers at FOF1), repeated measures of hand grip strength (among mothers only, at FOM2, FOM3, FOM4), repeated measures of NMR metabolomics (among mothers at FOM1, FOM2 and among fathers at FOF1), one measure of insulin (among mothers at FOM1 and among fathers at FOF1), repeated measures of CRP (among mothers at FOM1, FOM2, FOM3, FOM4 and among fathers at FOF1), and

reported diagnosis of type 2 diabetes. Regression-based analyses will be done as above.

Exposures, outcomes and confounders to be considered (justifying particular types of data as necessary): Described above

Reasons for using ALSPAC: ALSPAC is uniquely suited for this study given the availability of repeated measures of body scanning (DXA)-based total and regional lean mass, repeated measures of grip strength (a rarity at younger ages), and repeated measures of detailed metabolic traits on both offspring and their parents, together spanning four key stages of life: childhood, adolescence, young-adulthood, and middle-adulthood. Metabolic trait outcomes are extensive, comprising novel NMR-derived traits and more established clinical traits. The young age of offspring participants is also an advantage as this allows associations to be examined in a generally healthy pre-clinical population which naturally boosts causal inference (in contrast to most prior studies that use samples of middle-aged or older adults only, wherein confounding by existing disease/reverse causation is a major concern).

What do you think the likely impact of your research will be?: The likely output of this research will be at least one publication in a general medical or epidemiology journal, the impact of which is expected to be theoretical advancement in active research fields of body composition and diabetes, and contributions towards more refined clinical and public health recommendations.

Subject classification (please select one): Epidemiology

Other subject - please specify:

----------------Diseases/conditions (click to expand/collapse)----------------
- **Please tick all appropriate diseases/conditions:** Diabetes

- **Other disease/condition - please specify:**

----------------Techniques (click to expand/collapse)----------------
- **Please tick all appropriate techniques:** Metabolomics

- **Other techniques - please specify:**

----------------Keywords (click to expand/collapse)----------------
- **Please tick all appropriate keywords:** Metabolic - metabolism

- **Methods - please specify:**

- **Other keyword(s) (please specify):**

Types of existing data required

Does your project involve analysing existing data?: Yes
Existing questionnaire data: Yes

Existing clinic data: Yes

Existing data from biological samples (not genetic): Yes

----------------Existing genome-wide SNP genotype data----------------
- **Existing genetics data:** No

----------------Existing Methylation Data----------------
- **Methylation data:** No

----------------Linkage data----------------
- **Third party data (e.g. data provided by education/health organisations):** No

----------------Address Data----------------
- **Do you require any data linked to addresses?:** No

----------------Text data----------------
- **Text data:** No

NEW data or sample collection

Are you requesting the collection of new data and/or biosamples?: No
----------------New data/biosamples----------------
- **Please name the person responsible for handling any incidental findings from the data / sample collection:**

- **Please provide details of what data you want to collect:**

- **How will your data be collected?:**

Processing of samples

----------------Do you want new genotyping carried out by LGC?----------------
- **SNP genotyping:** No

----------------Do you want new genotyping or methylation data generated on the Illumina platform?----------------

- **Do you require Illumina arrays to be run in the ALSPAC Laboratory?:** No

----------------Do you require DNA samples for other analysis?----------------
- **Do you require DNA samples to analyse elsewhere?:** No

Non-DNA biological samples

Will the project require access to biological samples other than DNA?: No

----------------Other biological samples----------------

Terms and Conditions

1. Please confirm that...: ...you are familiar with the latest version of the ALSPAC access policy (http://www.bristol.ac.uk/alspac/researchers/access)

2. Please confirm that...: ...you understand it is your responsibility to ensure that all members of your team working on this project complete a Data User Responsibilities Agreement (DURA) and that you inform ALSPAC of any changes to the team

3. Please confirm that...: ...you understand that data and samples from the ALSPAC resource cannot be used for commercial gain

4. Please confirm that...: ...you understand that you and your team must not pass on any data or samples awarded, or any derived variables or genotypes generated by this application to a third party (i.e. to anybody who is not included in the list of applicants on this form)

5. Please confirm that...: ...you aware that any third party seeking to use data, samples, or derived variables or genotypes arising from this application must approach ALSPAC to obtain access permission of their own?

6. Please confirm that...: ...you understand that if a problem arises involving any misuse of the ALSPAC data or samples provided for this project that violates any of the terms and conditions specified by the Data Access Agreement (DAA) that you (as principal applicant) have signed, you will be held responsible. This might result in you being excluded from using the ALSPAC resource in the future.

7. I understand that...: ...costs will be determined after the proposal has been approved and that I will not receive any data or samples until I have settled my invoice or provided a purchase order number.

8. I understand that...: ...all genotypes and/or data generated from biological

samples will be returned to ALSPAC and be made available to other researchers.

9. I declare that...: ...I have no conflict of interest in relation to this research.

If you do have a conflict on interest, please declare it here:

Date: 2019-03-20

Print name (this will serve as your signature): Joshua Bell
