# Supplementary material for: Body muscle gain and markers of cardiovascular disease susceptibility in young adulthood: A cohort study
Source: PLoS Med. 2021 Sep 9;18(9):e1003751. doi: 10.1371/journal.pmed.1003751 (PMC8428664; doi:10.1371/journal.pmed.1003751)
Supplement: S1 Table — Values are mean (SD) unless otherwise noted. Described are those without data on each lean, grip, and fat measure on any occasion, at least 1 cardiometabolic trait at 25 y, and each covariate used for models. *Change is from age 12 y to 25 y and is based on difference in SD units given the different original measurement units between occasions. (PDF) [file pmed.1003751.s013.pdf]

S1 Table Characteristics of 12,418 ALSPAC G1 offspring not eligible for present analyses

Characteristics	*N*	*Overall N=11,816*	*N*	*Males N=6,438*	*N*	*Females N=5,378*
Non-white ethnicity – % (N)	8913	5.5 (487)	4990	5.4 (269)	3908	5.5 (216)
Highest maternal education is degree – % (N)	9256	10.1 (933)	5182	10.2 (530)	4057	9.9 (400)
Age (y) at peak height velocity	2778	12.8 (1.3)	1526	13.6 (0.9)	1249	11.7 (0.8)
Smoking at 25y – % (N)	775		242		532	
Never		36.4 (282)		39.7 (96)		34.8 (185)
Less than weekly		58.5 (453)		57.0 (138)		59.2 (315)
Every week		5.2 (40)		3.3 (8)		6.0 (32)
Alcohol consumption at 25y – % (N)	748		232		515	
Never/monthly/less than monthly		32.0 (239)		27.6 (64)		33.8 (174)
2 to 4 times per month		41.4 (310)		40.1 (93)		42.1 (217)
2 or more times per week		26.6 (199)		32.3 (75)		24.1 (124)
Total limb lean mass index (kg/m^2) at 25y	732	7.2 (1.3)	234	8.2 (1.2)	497	6.7 (1.0)
Arm lean mass index (kg/m^2) at 25y	732	1.7 (0.4)	234	2.2 (0.4)	497	1.5 (0.2)
Leg lean mass index (kg/m^2) at 25y	732	5.5 (0.9)	234	6.0 (0.9)	497	5.2 (0.8)
Maximum grip strength (lb/in^2) at 25y	305	14.0 (3.8)	102	17.5 (3.8)	203	12.2 (2.4)
Relative grip strength (lb/in^2/kg) at 25y	300	0.2 (0.1)	101	0.2 (0.1)	199	0.2 (0.04)
Total fat mass index (kg/m^2) at 25y	732	8.7 (4.3)	234	6.6 (3.1)	497	9.7 (4.4)
Changes from childhood to young adulthood, 10y to 25y						
Total limb lean mass index (kg/m^2)	237	1.9 (1.1)	91	2.7 (1.1)	146	1.4 (0.8)
Arm lean mass index (kg/m^2)	237	0.6 (0.4)	91	1.0 (0.4)	146	0.3 (0.2)
Leg lean mass index (kg/m^2)	237	1.3 (0.8)	91	1.7 (0.8)	146	1.1 (0.6)
Maximum grip strength (SD)*	134	0.1 (1.1)	47	0.1 (1.0)	87	0.1 (1.1)
Relative grip strength (SD)*	132	0.1 (1.0)	47	0.2 (1.0)	85	0.1 (1.0)
Total fat mass index (kg/m^2)	237	3.8 (3.1)	91	2.8 (2.6)	146	4.4 (3.2)
Changes in childhood, 10y to 13y						
Total limb lean mass index (kg/m^2)	2690	1.0 (0.6)	1542	1.3 (0.6)	1143	0.7 (0.4)
Arm lean mass index (kg/m^2)	2690	0.3 (0.2)	1542	0.4 (0.2)	1143	0.2 (0.1)
Leg lean mass index (kg/m^2)	2690	0.7 (0.4)	1542	0.9 (0.4)	1143	0.5 (0.3)
Total fat mass index (kg/m^2)	2690	0.9 (1.8)	1542	0.5 (1.8)	1143	1.4 (1.6)
Changes in adolescence, 13y to 18y						
Total limb lean mass index (kg/m^2)	1552	0.5 (0.7)	843	0.9 (0.7)	708	-0.03 (0.4)
Arm lean mass index (kg/m^2)	1552	0.3 (0.3)	843	0.5 (0.2)	708	0.1 (0.1)
Leg lean mass index (kg/m^2)	1552	0.2 (0.5)	843	0.4 (0.5)	708	-0.1 (0.4)
Total fat mass index (kg/m^2)	1552	1.0 (2.1)	843	0.5 (2.0)	708	1.7 (1.9)
Changes in young adulthood, 18y to 25y						
Total limb lean mass index (kg/m^2)	306	0.6 (0.7)	102	0.5 (0.8)	204	0.7 (0.7)
Arm lean mass index (kg/m^2)	306	0.1 (0.2)	102	0.1 (0.3)	204	0.04 (0.2)
Leg lean mass index (kg/m^2)	306	0.5 (0.6)	102	0.3 (0.6)	204	0.6 (0.6)
Total fat mass index (kg/m^2)	306	1.7 (2.4)	102	2.2 (2.2)	204	1.4 (2.4)

Values are mean (SD) unless otherwise noted. Described are those without data on each lean, grip, and fat measure on any occasion, at least 1 cardiometabolic trait at 25y, and each covariate used for models. *Change is from age 12y to 25y and is based on difference in SD units given different original measurement units between occasions.
