# Supplementary material for: Body muscle gain and markers of cardiovascular disease susceptibility in young adulthood: A cohort study
Source: PLoS Med. 2021 Sep 9;18(9):e1003751. doi: 10.1371/journal.pmed.1003751 (PMC8428664; doi:10.1371/journal.pmed.1003751)
Supplement: S3 Table — (PDF) [file pmed.1003751.s015.pdf]

S3 Table Pearson correlations amongst changes in limb lean mass indices, total fat mass index, and grip strength, 12y to 25y

	Limb lean mass index, 12y to 25y	Arm lean mass index, 12y to 25y	Leg lean mass index, 12y to 25y	Maximum grip strength, 12y to 25y	Relative grip strength, 12y to 25y	Total fat mass index, 12y to 25y
Limb lean mass index, 12y to 25y	1.00	0.81	0.97	0.33	-0.12	0.39
Arm lean mass index, 12y to 25y	-	1.00	0.66	0.34	-0.07	0.30
Leg lean mass index, 12y to 25y	-	-	1.00	0.30	-0.13	0.39
Maximum grip strength, 12y to 25y	-	-	-	1.00	0.76	0.03
Relative grip strength, 12y to 25y	-	-	-	-	1.00	-0.44
Total fat mass index, 12y to 25y	-	-	-	-	-	1.00
