# Supplementary material for: Body muscle gain and markers of cardiovascular disease susceptibility in young adulthood: A cohort study
Source: PLoS Med. 2021 Sep 9;18(9):e1003751. doi: 10.1371/journal.pmed.1003751 (PMC8428664; doi:10.1371/journal.pmed.1003751)
Supplement: S4 Table — (PDF) [file pmed.1003751.s016.pdf]

S4 Table Pearson correlations between change in limb lean mass indices and total fat mass index, 10y to 13y

	Limb lean mass index, 10y to 13y	Arm lean mass index, 10y to 13y	Leg lean mass index, 10y to 13y	Total fat mass index, 10y to 13y
Limb lean mass index, 10y to 13y	1.00	0.83	0.98	0.09
Arm lean mass index, 10y to 13y	-	1.00	0.69	0.14
Leg lean mass index, 10y to 13y	-	-	1.00	0.06
Total fat mass index, 10y to 13y	-	-	-	1.00
