# Supplementary material for: Body muscle gain and markers of cardiovascular disease susceptibility in young adulthood: A cohort study
Source: PLoS Med. 2021 Sep 9;18(9):e1003751. doi: 10.1371/journal.pmed.1003751 (PMC8428664; doi:10.1371/journal.pmed.1003751)
Supplement: S5 Table — (PDF) [file pmed.1003751.s017.pdf]

S5 Table Pearson correlations between change in limb lean mass indices and total fat mass index, 13y to 18y

	Limb lean mass index, 13y to 18y	Arm lean mass index, 13y to 18y	Leg lean mass index, 13y to 18y	Total fat mass index, 13y to 18y
Limb lean mass index, 13y to 18y	1.00	0.74	0.97	0.11
Arm lean mass index, 13y to 18y	-	1.00	0.57	0.12
Leg lean mass index, 13y to 18y	-	-	1.00	0.09
Total fat mass index, 13y to 18y	-	-	-	1.00
