# Supplementary material for: Body muscle gain and markers of cardiovascular disease susceptibility in young adulthood: A cohort study
Source: PLoS Med. 2021 Sep 9;18(9):e1003751. doi: 10.1371/journal.pmed.1003751 (PMC8428664; doi:10.1371/journal.pmed.1003751)
Supplement: S7 Table — (PDF) [file pmed.1003751.s019.pdf]

S7 Table Pearson correlations amongst limb lean mass indices, total fat mass index, and grip strength at 25y

	Limb lean mass index, 25y	Arm lean mass index, 25y	Leg lean mass index, 25y	Maximum grip strength, 25y	Relative grip strength, 25y	Total fat mass index, 25y
Limb lean mass index, 25y	1.00	0.84	0.98	0.31	-0.33	0.59
Arm lean mass index, 25y	-	1.00	0.70	0.38	-0.09	0.32
Leg lean mass index, 25y	-	-	1.00	0.26	-0.39	0.65
Maximum grip strength, 25y	-	-	-	1.00	0.66	-0.01
Relative grip strength, 25y	-	-	-	-	1.00	-0.62
Total fat mass index, 25y	-	-	-	-	-	1.00
