# Supplementary material for: Body muscle gain and markers of cardiovascular disease susceptibility in young adulthood: A cohort study
Source: PLoS Med. 2021 Sep 9;18(9):e1003751. doi: 10.1371/journal.pmed.1003751 (PMC8428664; doi:10.1371/journal.pmed.1003751)
Supplement: Attachment — Submitted filename: Response to Reviewers.docx [file pmed.1003751.s045.docx]

**Response to Reviewers**

We thank the editors and reviewers for constructive comments on our manuscript, titled **‘Body muscle gain and markers of cardiovascular disease susceptibility in young adulthood: prospective cohort study’**. These comments are copied below, followed by our responses.

**Reviewer 1**

*1. I confine my remarks to statistical aspects of this paper. Unfortunately, I think some major changes need to be made. The biggest is that, rather than look at change in the dependent variables, the authors should use either a multilevel model or generalized estimating equations to deal with the dependent errors.*

RESPONSE: To clarify, the metabolic trait outcomes (dependent variables) examined in this study are measured at a single time point, at age 25y, and differences in levels of these are examined in relation to prospective change in each lean/fat mass exposure trait (independent variables) across several different time points. Mixed modelling approaches were not deemed preferable to more basic linear regression modelling here, however, given that our exposures were examined over different life stages (childhood/pre-puberty, adolescence/post-puberty, and young adulthood) which require occasion-specific confounding adjustments which were more feasible with basic regression models. Mixed modelling would also not be feasible with handgrip strength exposures given that only 2 measures are available, and basic regression models enable us to generate estimates which are directly comparable across all exposures (lean mass indices, fat mass index, grip strength).

This point does not specify any other statistical concerns and our general approach to modelling was supported by 2 other reviewers. Several concerns about measurement units and statistical adjustments are mentioned by other reviewers and we outline such edits below.

*2. p. 3 "strong cause" .... has causation been estatblished here?*

RESPONSE: Thank you; ‘strong cause’ has been softened to ‘likely cause’ here. We base causal inference on principles of triangulation and this statement is based on consistent estimates of effect from prospective observational and Mendelian randomization studies, and from human mechanistic evidence from basic biology studies on the glycemic and inflammatory effects of adipose tissue. References for these different study types are made in the first introductory paragraph on **page 4**.

*3. why look at total lean mass? Surely someone who is 2 meters tall will have a lot more mass than someone who is 1.5 m. tall.*

RESPONSE: We do not examine total lean mass in this study. Total lean mass would indeed vary by height, as would total fat mass; we therefore examine total fat mass in this study as an index of squared height to account for variation by height, as is most commonly done when estimating effects of total fat mass indirectly via body mass index (in kg/m^2^).

*4. Handgrip as an indicator of overall strength seems a bit problematic too. Handgrip only tests strength in one muscle.*

RESPONSE: As mentioned by Reviewer 3, handgrip strength is a direct measure of strength in one isolated muscle group but a widely used indirect measure of overall muscle strength given its high positive correlation with more objectively measured muscle compartments. We note this in the second introductory paragraph on **page 5** with added references as follows:

“Benefits of muscle may also be reflected in strength which can be measured directly for isolated arm muscles using handgrip tests; grip strength correlates well (>0.7) with more objectively measured strength in other muscle groups such as hips and is thus a useful and widely used proxy for overall muscular strength in larger scale studies (14-16).”

We initially examined grip strength as a function of total fat mass index but following suggestions from Reviewer 3, we have updated grip strength analyses to reflect grip divided by body weight in kg, as this should better correct for overall body size rather than fat mass itself (which is not the intention here) and reduce the potential for results for grip strength to be influenced by its negative correlation with fat mass. This also enables consistent adjustment for total body fat mass as a confounder in multivariable regression models as well as more consistent effect estimates based on future Mendelian randomization studies, given that GWAS of grip strength have corrected for weight rather than body mass index or fat mass index.

*5. "No protection" do you mean no significant protection? Give effect sizes.*

RESPONSE: Thank you; we agree that effect sizes are informative here and these have been added into the introduction on **page 5** as follows:

“Prospective observational estimates suggest that higher limb lean mass and stronger grip are both associated with lower CHD risk independent of body mass index (BMI) (14, 17, 18), and factorial Mendelian randomization estimates based on externally weighted genetic instruments suggest that the risk ratio for CVD onset is comparable among adults with both high grip and high BMI (1.04, 95% CI = 0.98, 1.11) as among adults with both low grip and high BMI (1.03, 95% CI = 0.97, 1.10), compared with adults with both high grip and low BMI (P-value for interaction = 0.50) (19).”

These estimates are based on factorial Mendelian randomization using individual level data from the UK Biobank study and binary categorisations of BMI and grip strength; such categorizations are not ideal but continuous estimates are not available and we consider effect sizes to be informative here in addition to a P-value for interaction (P=0.50). We quote estimates from models which used external weightings for genetic instruments because these are considered less prone to ‘winners curse’ biases from overlap with discovery and outcome samples (see *Haycock et al. Best (but oft-forgotten) practices: the design, analysis, and interpretation of Mendelian randomization studies. Am J Clin Nutr. 2016*).

*6. p. 6. Lumping all nonwhites together seems arbitrary and likely to increase error.*

RESPONSE: The ALSPAC Generation-1 offspring cohort are approximately 95% white-British, which leaves very few participants in sub-categories of the non-white group for meaningful grouping and inference. This crude broader grouping is a common trade-off between measurement error and imprecision from low sample size. Given that overall sample size is already relatively modest, we elected to retain this binary ethnicity grouping, as most commonly done in ALSPAC studies. Ethnicity is examined here for the purposes of sample description and confounder adjustment; it is not an aim of this study to examine whether effects of exposures on outcomes differ by ethnicity.

*7. Independent variables like alcohol consumption should not be categorized. See Frank Harrell "Regression modeling strategies"*

RESPONSE: We do not have the option of analysing independent variables that are currently in categorical form, such as alcohol consumption and smoking behaviour, as continuous variables because they were not originally measured as such; these data are collected in categorical form. These variables are used for the purposes of sample description and confounder adjustment only; it is not the aim of this study to examine whether effects of exposures on outcomes differ by factors other than sex. One advantage of analysing offspring participants at young ages is the relative rarity or recency of onset of adverse behaviours such as smoking and alcohol consumption, which should reduce their potential to act as strong confounders of the exposure-outcome effects being estimated. We mention this in the limitations section on **page 19**.

**Reviewer 2**

*1. It is an elegant and exhaustive paper that shows little added value to consider muscle / lean mass trajectories during childhood, compared to fat mass, as a risk factor for atherogenic profile in young adulthood. The research question is nicely introduced, and the analyses seem thorough. I have two main concerns regarding the analyses that will need to be addressed though: 1) account for multiple testing; 2) unrestricted sample analysis is not a clean way to compare estimates as the samples are varying from trait to trait / exposure to exposure. Also overall the conclusions are not supported by the results and should be toned down. My specific comments are detailed below:*

*Abstract: "and others" should be removed or specified.*

RESPONSE: Thank you; ‘and others’ has been removed in the abstract on **page 2**, as well as in the methods on **pages 9-10**.

*2. [Abstract:] "further specific"?*

RESPONSE: This has been changed to “Furthermore, this pattern for arm lean mass index was specific to…” in the abstract on **page 2**.

*3. [Abstract:] Conclusion first sentence should be rephrased.*

RESPONSE: The first sentence of the conclusion is based on results from grip strength, not lean mass indices, and we consider this to accurately reflect the pattern of results. We have expanded this sentence for clarity on **page 3** as follows:

“Muscle strengthening, as indicated by grip strength gain, is weakly associated with lower atherogenic trait levels in young adulthood, at a smaller magnitude than unfavourable associations of fat mass gain.”

We now specify “muscle mass gain” in the sentence immediately following this.

*4. Intro: "… while Mendelian randomization estimates suggest no protection from stronger grip independent of BMI (16)." Here you probably need to highlight that there is no Mendelian randomization on limb lean mass?*

RESPONSE: Thank you; this is a useful addition and we have revised this sentence, additionally in response to Reviewer 1, on **page 5** as follows:

“Prospective observational estimates suggest that higher limb lean mass and stronger grip are both associated with lower CHD risk independent of body mass index (BMI) (14, 17, 18), and factorial Mendelian randomization (MR) estimates suggest that the risk ratio for CVD onset is comparable among adults with both high grip and high BMI (1.04, 95% CI = 0.98, 1.11) as among adults with both low grip and high BMI (1.03, 95% CI = 0.97, 1.10), compared with adults with both high grip and low BMI (P-value for interaction = 0.50) (19). Such factorial MR estimates for limb lean mass, whether based on bioimpedance or more precise dual-energy x-ray absorptiometry (DXA) scans, are not yet available.”

*5. " Males have higher lean mass and stronger grip than females (13, 17), but whether these are more cardiometabolically beneficial among males is unknown." This sentence is somewhat paradoxical. Males at higher risk of CVD/CHD than women, so having more lean mass does not appear to be potentially protective. And why would lean mass / strength be more cardiometabolically beneficial among males than among women? You are talking about strength of association? This justification of the exploration of the interaction with sex is a bit poor and would need to be developed.*

RESPONSE: Thank you; we agree that this needs more explanation. Our examination of sex differences here is motivated by previous evidence on sex differences in the associations of fat mass with these same atherogenic traits measured using targeted metabolomics; previous studies suggested more adverse patterns of association among males than females, with these sex differences in turn varying by life stage. These sex differences have not been previously examined for muscle strength or muscle mass, and our approach here is more agnostic such that we present results in their entirety in supplementary files. We have expanded our rationale for this in the introductory section on **page 6** as follows:

“Growing evidence suggests that associations between body fat mass and atherogenic traits differ importantly by sex. Higher fat mass, whether measured using BMI, waist circumference, or DXA fat mass, appears to be more strongly positively associated among males with CVD-relevant traits including metabolomics-derived glucose, apolipoprotein-B lipids, and inflammatory glycoprotein acetyls (GlycA) (7, 28); these sex differences in turn vary by stage of body development with associations appearing more adverse among males in childhood, adolescence, and young adulthood, but similar or more adverse among females in middle adulthood (28). Such differences do not simply reflect differences in total fat volume since males tend to carry less total fat than females throughout life (28, 29); this instead suggests an important role of abdominal and ectopic fat storage, which is often higher among males (4, 29), in underpinning the effects of total fat (10). Males tend to have higher lean mass and stronger grip than females (14, 30), but whether important sex differences exist in the associations of muscle mass or strength with atherogenic traits is unknown. Sex differences in the potential benefits of muscle have also not been previously examined in relation to detailed atherogenic traits measured from targeted metabolomics (31). Muscle tissue is thought to be more modifiable after childhood (32) and thus greater benefits from a higher contractile capacity may be expected from adolescence onwards; but the common lack of repeated measures of body muscle at different life stages has prevented examination of the potential modifying role of growth and development in the associations of muscle change with atherogenic traits (33).”

*6. Throughout the manuscript: every time you use "these traits" it is somewhat imprecise, I think it would be better to use "atherogenic traits" throughout.*

RESPONSE: Thank you; the only instance where the term “these traits” is used is in the last paragraph of the introduction, and we have updated this on **page 6** to “atherogenic traits” as suggested.

*7. Methods: Confounder: ideally, alcohol and smoking would be measured at time of exposure. Was this question not asked at earlier visits than at 25y?*

RESPONSE: Thank you, and apologies: this was meant to read ‘18y’ as these smoking and alcohol were measured and adjusted for at the same time as exposures assessed from age 18y. Because we have added cross-sectional analyses in response to another reviewer comment, we adjust for smoking and alcohol measured at age 25y in those additional models. This has been clarified in the methods section on **pages 9-10**.

These behaviours are not yet measured at age 10y or 13y. Prevalence of these behaviours at those ages is expected to be very low and if present, of very recent onset such that they are not yet expected to substantially influence cardiometabolic trait levels.

*8. I have concerns on the unrestricted sample analysis with N varying, as the sample selection makes for each trait / time period is likely to making results not comparable. I suggest the complete case be the main analysis, at least with available data at 10y and 25y.*

RESPONSE: We appreciate this concern; due to the large number of exposure and outcome measures and long periods of follow-up used for this study, the complete case sample is greatly reduced from those who were initially eligible, from 3,227 to 770 participants. To enable greater estimate precision, we base main analyses and figures on unrestricted samples of participants (who have data on the variables needed for individual models being run, allowing Ns to vary), and have repeated all models based on the complete-case sample so that all estimates can be compared (all are given in Supp Tables). These complete-case estimates are expectedly less precise due to their smaller sample size, but the direction and magnitude of results are very similar. For example:

Effect estimate of change in limb lean mass index from age 10y to 25y on VLDL triglycerides:

Unrestricted N=2295; 0.17 SD, 95% CI=0.10, 0.24

Complete-case N=770; 0.20 SD, 95% CI=0.10, 0.30

Effect estimate of change in limb lean mass index from age 10y to 25y on GlycA:

Unrestricted N=2295; 0.17 SD, 95% CI=0.09, 0.24

Complete-case N=770; 0.20 SD, 95% CI=0.11, 0.29

Effect estimate of change in relative grip strength from age 12y to 25y on VLDL triglycerides:

Unrestricted N=1371; -0.12 SD, 95% CI=-0.18, -0.06

Complete-case N=770; -0.12 SD, 95% CI=-0.20, -0.04

Effect estimate of change in relative grip strength from age 12y to 25y on GlycA:

Unrestricted N=1371; -0.13 SD, 95% CI=-0.18, -0.07

Complete-case N=770; -0.11 SD, 95% CI=-0.19, -0.03

Effect estimate of change in fat mass index from age 10y to 25y on VLDL triglycerides:

Unrestricted N=2295; 0.45 SD, 95% CI=0.39, 0.52

Complete-case N=770; 0.44 SD, 95% CI=0.33, 0.56

Effect estimate of change in fat mass index from age 10y to 25y on GlycA:

Unrestricted N=2295; 0.54 SD, 95% CI=0.48, 0.60

Complete-case N=770; 0.48 SD, 95% CI=0.38, 0.58

*9. Did you test for interactions with sex if you stratified your results by sex and seem to have a strong hypothesis that the associations might be different for males vs females*

RESPONSE: We do not formally test for statistical interaction between sex and each exposure, using product terms, in relation to each outcome trait examined here. We appreciate that this step can be useful for providing additional evidence of the degree of interaction present in a given sample, but we elected not to conduct these for several reasons:

1. Our present sample sizes are relatively modest for this purpose and interaction tests generally require large sample sizes for adequate power, e.g., the P-value for interaction between BMI and grip strength in relation to CVD from factorial Mendelian randomization analyses quoted in the introduction section is based on samples of over 100,000 adults.
2. The volume of analyses makes these tests practically unfeasible. Following reviewers’ requests for additional analyses, sex-combined analyses currently involve 26 variants of exposure variables, each with 228 cardiometabolic trait outcomes (~5,900 effect estimates for sexes combined). Adding P-values for interaction tests for each of these pairings would require an additional 26 columns of P-values in current supplementary files.
3. It is unclear on what basis results would be stratified or not by sex. We note in response to point 10 below about multiple testing corrections that we aim to base interpretations of results on effect size and precision, not arbitrary thresholds based on P-values which are known to be highly sensitive to sample size and data derivations. Relying on such interaction P-value thresholds here would be an inconsistent approach and inevitably lead to some estimates apparently warranting stratification while others do not; this alone would lead to very difficult presentations and interpretations of results given the high volume at hand.

There is a strong rationale based on previous evidence for examining associations of fat mass with cardiometabolic traits separately by sex. Much less has been previously done on muscle exposures in relation to these traits, and we aimed to examine sex differences for each muscle exposure in a comparable way to that done for fat mass, agnostically with the intention of reporting results in full in supplementary files. We consider this approach to be more transparent and less prone to bias from selective reporting.

*10. If you looked at 145 concentrations and 79 ratios of metabolomics markers, you should account for multiple testing in some way. It is unclear if you tested all of the markers of if you did some preselection.*

RESPONSE: The metabolic traits examined as outcomes, besides blood pressure, were measured using a single high-throughput platform for NMR targeted metabolomics. We elected to analyse all measures from this platform and present results in full given that these traits are biologically inter-related and many have not been previously examined in relation to these exposures. We also consider this approach to be more transparent and less subject to bias from selective reporting.

We used P-values within an estimation framework for this study, as continuous indicators of the strength of evidence against null hypotheses of no difference. P-value thresholds can be useful within a discovery framework when there is a need to prioritise factors with the strongest evidence in a given sample so that a more limited set can be taken forward into a next stage of analysis (such as in a genome-wide association study); but relying on these thresholds for inference for estimation purposes is actively discouraged by the American Statistical Association (ASA) (*Wasserstein & Lazar. 2016. The ASA’s statement on p-values: context, process, and purpose. Am Statistician. 70(2):129-33*) and others (*Sterne & Davey Smith. Sifting the evidence—what's wrong with significance tests? BMJ. 2001*). Reliance on P-value thresholds (‘significant or not’) in most instances is discouraged because P-values are known to be highly sensitive to sample size and data derivations, and because this helps to avoid imposing false dichotomies on chance. For example, a P-value above 0.05 does not mean that a result *is* due to chance, and a P-value below 0.05 does not mean that a result *is not* due to chance. Multiple testing corrections necessarily rely on such thresholds and we do not consider them meaningful or relevant to current study aims. For example, there would be no difference between these results presented in a single paper and these results presented across many separate papers; multiple testing would not be a concern in the latter scenario, despite exactly the same analyses and data.

In line with ASA recommendations, we focus interpretations of results on effect sizes and precision via 95% confidence intervals and provide exact P-values in supplementary tables (i.e., not simply ‘P less than’ some threshold). If others wish, the exact P-values provided can be used to penalise individual estimates against a chosen threshold that represents the number of independent tests performed (there is unlikely to be consensus on this number), noting expectedly high correlations between metabolites.

*11. Results: Ineligible vs included participants. Nowhere in the Method section is the explanation of who are the ineligible participants. Please insert the information from the flow diagram Figure 1 into the Method section.*

RESPONSE: Thank you; we agree that this description is needed and have added a paragraph in the methods section on **page 8** as follows:

“We conducted main analyses on unrestricted samples of participants (N varying between traits and across occasions) to enable use of all measured data. Of the 14,901 surviving Generation-1 ALSPAC participants who were eligible for future clinic assessments, 6,119 participants had data on covariates used for model adjustments plus ≥ 1 of any of: DXA measures (lean or fat mass) or grip strength at age 25y; or change in DXA measures across the total observation period (10y to 25y), across childhood (10y to 13y), across adolescence (13y to 18y), or across young adulthood (18y to 25y); or change in grip strength across the total observation period (12y to 25y). Of those 6,119 participants with any exposure measure, 3,227 participants had data on ≥ 1 cardiometabolic trait at age 25y and were thus considered eligible for inclusion in at least one of the present set of analyses (**Figure 1**).”

*12. The paragraph "Correlations amongst changes in lean mass indices, grip strength, and fat mass index" does not flow easily, please try and rephrase.*

RESPONSE: Thank you; we have updated this paragraph in the results section on **page 12** as follows:

“Change in limb lean mass index between ages 12y and 25y was positively correlated with change in maximum grip (r = 0.33) but negatively correlated with change in relative grip (r = -0.12) over this same period. Change in limb lean mass index was positively correlated with change in fat mass index over this same period (r = 0.39), as well as between ages 10y and 25y (r = 0.47). Change in limb lean mass index was more positively correlated with change in leg lean mass index than change in arm lean mass index, based on either time period (**Supp Tables 2-3**). Based on changes occurring between ages 12y and 25y, fat mass index change was uncorrelated with maximum grip change (r = 0.03) but negatively correlated with relative grip change (r = -0.44). Considering different sub-periods, change in fat mass index was more positively correlated with change in arm lean mass index than change in leg lean mass index over childhood and adolescence (Supp Tables 4-5), whereas change in fat mass index was more positively correlated with change in leg than arm lean mass index in young adulthood (Supp Table 6). A similar correlation pattern was seen cross-sectionally based on measures taken at age 25y (**Supp Table 7**).”

*13. Page 10 Associations of change in grip strength with cardiometabolic traits Please state in the sentence the direction of the association with VLDL triglycerides*

RESPONSE: Thank you; this has been updated in the results section on **page 14** as follows:

“Change in maximum grip from 12y to 25y (per SD-unit gain) was positively associated with creatinine but associations were largely null with atherogenic traits including VLDL triglycerides (at -0.01 SD, 95% CI = -0.07, 0.04; Figure 5; Supp Table 19), among males and females (Supp Tables 20-21).”

*14. Discussion: The summary page 11"suggest that muscle strengthening is associated with lower atherogenic trait levels" (also found in the abstract) does not reflect accurately the results, as the associations of change in limb lean mass from 10y to 25y with most atherogenic traits were of modest magnitude and mostly in directions assumed to reflect poorer health. Please tone down this sentence. This goes for the last paragraph of the discussion and for the abstract as well.*

RESPONSE: Thank you; this statement about muscle strengthening is based on results from grip strength, not lean mass indices, and we consider this to accurately reflect the pattern of results. We have added “as indicated by grip strength gain, was weakly associated with…” to clarify this in the abstract conclusion as well as in the discussion section on **pages 16 and 20**.

*15. Page 12, I do not understand the phrase "phenotypic features of genetically liability to adult CHD".*

RESPONSE: Thank you; this is meant to describe results of previous analyses using the same ALSPAC offspring data which examined associations of a genetic risk score for adult CHD with these same metabolomic traits, the aim being to identify metabolites which are most reflective of disease susceptibility/liability. We have updated this sentence on **page 16** as follows:

“The cardiometabolic traits considered here include several lipoproteins which previous ALSPAC analyses suggested are positively associated with a genetic risk score for adult CHD; higher levels of these traits are thus taken to reflect greater CHD liability (42).”

*16. Page 12. I also do not understand the logic in the last sentence of the paragraph "Change in leg lean mass may also better reflect body shape and abdominal fat storage, the compartment driving effects of total fat". Are you saying that leg lean mass gain is a proxy for total fat gain? Why is that? Can you please expand?*

RESPONSE: Thank you; upon review, we agree that this statement is not correct. This was likely originally referring to leg fat mass, rather than leg lean mass; with higher levels of fat stored in legs potentially indicating a higher proportion of fat stored peripherally rather than abdominally. Leg fat is not relevant to study aims and we have removed this sentence.

*17. Page 12. The following paragraph is somewhat empty. I think one could say that the fact that the relative index is more strongly associated may be reflecting simply the strong association with fat mass?*

RESPONSE: Thank you; we have updated all results involving relative grip strength after having re-derived the measure using ‘maximum grip / weight in kg’ instead of using fat mass index as prior. This is to better scale for overall body size rather than fat mass and to reduce potential for bias from an induced negative correlation with fat mass as mentioned. This also enables consistent adjustment for fat mass index as a confounder in multivariable models and enables consistent estimates of effect with future Mendelian randomization analyses which would likely use existing GWAS that corrected for weight instead of fat mass index or BMI.

The paragraph mentioned here has been updated in the discussion section on **page 17** to reflect these changes as follows:

“Associations of muscle strengthening with atherogenic traits were highly dependent on the grip measure used. Change in grip measured in absolute units (maximum grip) was positively associated with creatinine but generally unassociated with atherogenic traits, with point estimates close to null values of no difference. In contrast, change in grip measured in relative units (as a function of body weight) was also positively associated with creatinine but generally weakly associated with atherogenic traits in directions assumed to indicate better health, e.g. lower apolipoprotein-B lipids, glycemia, and inflammation. The magnitude of these associations of change in relative grip was higher than seen for arm lean mass index but generally lower than magnitudes seen for fat mass index, particularly among males where evidence was strongest. Furthermore, associations of higher maximum and relative grip strength with atherogenic traits were even less evident when assessed cross-sectionally in young adulthood, with point estimates close to null values. Considered alongside results on lean and fat mass indices, this supports body muscle as less robustly associated with cardiometabolic health in young adulthood than body fat, with a lower anticipated impact on CVD susceptibility from its modification.”

**Reviewer 3**

*1. The role of muscle mass and muscular strength in risk of cardiovascular and metabolic disease is gaining increasing interest. This study aimed to determine the associations between changes in these variables (as well as changes in adipsosity variables) from adolescences to young adulthood and cardiometabolic traits at age 25. The paper is well written and the analyses have been comprehsively undertaken. I do however have a few concerns that the authors should address:*

*It is unclear why only change in grip strength, lean mass and fat mass between adolescence and young adulthood was considered here, and not the actual values for these variables at age 25. Changes occuring over the course of maturation and puberty may not have the same effect as changes in these variables over the course of adulthood, so interpretation of these findings as to the importance of lean mass and muscular strength in affacting cardiometabolic traits is uncertain. The authors need to provide more justification about what assessing the change in these variables over time adds over and above just considering these variables at age 25. They should comment on whether you can extrapolate these changes to to other stages of life in the discussion. Could the authors also undertake the analyses using the values of grip strength, lean mass and fat mass variables at age 25 as the predictor variables? It would helpful to compare the results of these two analyses and comment as approriate in the discussion.*

RESPONSE: Thank you; we agree that further explanations are needed here. Our interest in change in fat/muscle exposures stems from earlier work suggesting consistent associations of change in fat mass between childhood and young adulthood with these same metabolomic traits measured at age 18y (*Bell et al. Associations of body mass and fat indexes with cardiometabolic traits. J Am Coll Cardiol 2018.*) and recent work suggesting substantial sex differences in associations of adiposity with metabolomic traits at different life stages, considering childhood to middle adulthood (*O’Keeffe et al. Sex-specific associations of adiposity with cardiometabolic traits: multi-life-stage cohort study with repeat metabolomics. medRxiv. 2020.)* The metabolomic profile of muscle exposures has not been previously examined, and we aimed to examine this in a consistent way as done for fat mass to compare the pattern and magnitude of associations. These have also not been previously examined in early life, and we aimed to examine these holistically and report results in full. We have expanded on this in the introduction on **page 6** and note the relevance of results to older age groups in the discussion’s limitations section on **page 19** as follows:

“In addition to current exposure levels, our analyses were based on changes in body muscle measures occurring early in life in relation to cardiometabolic traits measured in young adulthood (age 25y). These included an observation period of young adulthood (age 18y to 25y) which should be relevant to adulthood more broadly, but results based on changes occurring over childhood and adolescence (periods of growth and development with pronounced hormonal activity) may not represent the impact of changes occurring later in life. Muscle mass and strength may plausibly be more influential for CVD susceptibility at older ages when CVDs commonly start to emerge; obtaining unbiased estimates of effect in middle-to-later adulthood is difficult, however, because of increased potential for residual confounding (reverse causation). Other study designs such as MR may prove useful here.”

We also agree that there is value in additionally including cross-sectional analyses of each exposure in relation to cardiometabolic traits, to compare association profiles of prior changes in exposures with those of current levels of exposures. These analyses have been added into the paper (**Supp Tables 25-31**). These results suggest a higher level of consistency between prospective and cross-sectional assessments of fat mass index than of lean mass indices or grip strength, which appear weaker based on cross-sectional assessments.

These are mentioned in the methods section on **page 11** as follows:

“Lastly, following peer review, we examined cross-sectional associations of lean mass indices, grip strength, and fat mass index with cardiometabolic traits, all measured at 25y, to compare the association profile of changes in exposures to that of current levels of exposure. These models were adjusted for the same covariates as in models with exposure change measured starting from 18y, but with smoking, alcohol, and lean/fat mass indices now measured at 25y.”

And reported in the results text on **pages 14-15** as follows:

“*Cross-sectional associations of lean mass indices, grip strength, and fat mass index with cardiometabolic traits in young adulthood*

Evidence was weaker for cross-sectional associations of higher limb lean mass (per SD) with atherogenic lipid, glycemic, and inflammatory traits, e.g., -0.04 SD (95% CI = -0.10, 0.01) for apolipoprotein-B (**Supp Table 25**). Also, in contrast to estimates based on change in exposure, estimates appeared more favourable for per-SD higher leg lean mass index, e.g., apolipoprotein-B was -0.10 SD (95% CI = -0.18, -0.01) lower, yet 0.05 SD (95% CI = -0.02, 0.11) higher, for leg lean and arm lean mass indices, respectively. Leg lean mass index was more positively associated with creatinine than was arm lean mass index (point estimates were 0.21 SD versus 0.13 SD, respectively). These associations were more pronounced among females than males; despite similarly positive associations of lean mass indices with creatinine between the sexes (**Supp Tables 26-27**).

Evidence was substantially weaker or null for cross-sectional associations of both grip strength measures (per SD) with atherogenic lipid, glycemic, and inflammatory traits, e.g., 0.01 SD (95% CI = -0.05, 0.07) for apolipoprotein-B with stronger relative grip (**Supp Table 28**). Associations were more evident among males but with estimates appearing positive in relation to VLDL lipids, e.g., 0.10 SD (95% CI = -0.02, 0.22) for total lipids in very large VLDL (**Supp Tables 29-30**). Maximum grip was more positively associated with creatinine than was relative grip, among both sexes.

In contrast, the direction and magnitude of cross-sectional associations of higher fat mass index with atherogenic traits were highly comparable to those from previous models of change in exposure (**Supp Table 31**). For example, higher fat mass index (per SD) was associated with 0.42 SD (95% CI = 0.36, 0.48) higher triglycerides in VLDL, 0.47 SD (95% CI = 0.41, 0.53) higher insulin, and 0.53 SD (95% CI = 0.48, 0.58) higher GlycA. Estimates for most traits were comparable between the sexes, except for non-HDL lipids which were more positive among males (**Supp Table 31**).”

These are also mentioned in the discussion section across **pages 16-17**.

Because these cross-sectional analyses involved model adjustments for smoking and alcohol measured at age 25y (new variables considered), these additional analyses changed the sample size of our eligible sample (defined for the purpose of restricting descriptive characteristics tables, correlation analyses, and histograms) from 3,262 to 3,227. This also reduced our complete case sample from 787 to 770. Descriptive characteristics, correlations, histograms, and all complete-case analyses have been updated to reflect this change.

*2. It is unclear why the authors chose to express grip strength relative to fat mass index in particular. Please justify this. Why not relative to body weight, or height, or BMI or lean mass index? All of these relative indices have limitations and the authors need to be clear why they have chosen to express the findings in this way. Some of the results where there was an inverse relationship between relative but not absolute grip stength and atherogenic traits (e.g. for VLDL trigycleride) is probably driven by the the inverse relationship between fat mass and VLDL-TG, rather than the association with grip strength per se. The authors should consider this carefully and perhaps express relative grip strength relative to body weight or height. There are publications which have addressed the best approaches to normalise grip strength and the authors would be advised to consult these and justify their approach here.*

RESPONSE: Thank you; upon review, we agree that it would be more appropriate to scale maximum grip strength to overall body weight than to fat mass index. The purpose of this scaling was to account for expected differences in grip strength with body size, and not with fat mass specifically, and to provide a side-by-side comparison for maximum grip given the oftentimes stronger associations of relative grip with clinical outcomes. Deriving relative grip based on ‘maximum grip / weight’ also has the advantage of enabling consistent adjustment across measures for fat mass index as a confounder (this was not adjusted for previously because fat mass index was already used to derive the relative grip measure). This also has the advantage of being comparable to the phenotype used in a recent genome-wide association study of relative grip strength in UK Biobank (*Tikkanen et al. 2018. Biological Insights Into Muscular Strength: Genetic Findings in the UK Biobank. Sci Reports)*, enabling greater comparability between our observational estimates and future Mendelian randomization estimates.

Analyses of relative grip strength have been updated to reflect this; these new results are directionally consistent with previous estimates with more pronounced associations for relative grip than for maximum grip, but these new associations (now adjusted for fat mass index as a confounder) appear weaker than before, with smaller effect sizes. We also use the term ‘maximum grip’ instead of ‘absolute grip’ throughout the paper for simplicity.

Descriptions of this analysis have been updated in the methods section on **page 9** as follows:

“Maximum grip strength was estimated as the mean of three measures. Grip strength as a function of body weight, here termed ‘relative grip’, was calculated as maximum grip divided by body weight in kg. We scaled for weight rather than fat mass index or BMI when deriving relative grip following peer review; this was considered preferable because this: 1) accounts for overall body size rather than adiposity specifically; 2) reduces bias from an induced negative correlation between relative grip strength and adiposity; 3) enables consistent multivariable adjustment for fat mass index as a confounder across muscle measures; and 4) enables comparable estimates of effect with MR analyses given that genome-wide association studies have scaled for weight rather than BMI (42).”

And on **page 11**:

“Associations of change in grip measures from 12y to 25y with traits at 25y were examined with adjustment for basic demographic factors, grip strength at 12y, and change in fat mass index.”

Results have been updated in **Supp Tables 19-21** and in **Figure 5**.

The results text has been updated on **page 14** as follows:

“Change in maximum grip from 12y to 25y (per SD-unit gain) was positively associated with creatinine but associations were largely null with atherogenic traits including VLDL triglycerides (at -0.01 SD, 95% CI = -0.07, 0.04; **Figure 5; Supp Table 19**), among males and females (**Supp Tables 20-21**). In contrast, change in relative grip over the same period was positively associated with creatinine and moderately inversely associated with atherogenic traits, e.g. VLDL triglycerides at -0.12 SD, 95% CI=-0.18, -0.06, with similar magnitudes for apolipoprotein-B, insulin, GlycA, SBP, and DBP. These appeared strongest among females.”

*3. On page 4, the authors state that the causality of body muscle for CVD risk can be interogated by examining the assocations with key intermediates. I don't agree with this. Although the analyses will show correlations between muscle/strength and risk traits, it is entirely possible that this could be driven by confounders in this type of analysis. This statement should be tempered.*

RESPONSE: This statement about interrogating causality is meant to refer to the causal intent or motivation of analysis, rather than the assumed establishment of causality in results (as discussed in *Hernán 2018. The C-Word: Scientific Euphemisms Do Not Improve Causal Inference From Observational Data. Am J Public Health*). We have tempered and expanded this on **page 5** as follows:

“The likely causality of body muscle for CVD susceptibility can be interrogated by examining its associations with intermediate traits that have triangulated evidence of causality for CVD, in samples which have reduced potential for confounding.”

*4. Abstract, page 2, line 6. This sentence is not clear - is there a word or a comma missing?*

RESPONSE: Thank you; we assume this refers to the sentence beginning “Limb lean and total fat mass indices (kg/m^2^) were from…” on the second line of ‘Methods and Findings’. We have updated this to “Limb lean and total fat mass indices (kg/m^2^) were measured using…” in the abstract on **page 2**.

*5. Page 3, four lines up from bottom. A handgrip test provides a direct measurement of grip strength, so it is not correct to say that this in an indirect measure. You can say that grip strength is highly correlated with strength in other muscle groups so can provide a reasonable proxy for overall muscular strength.*

RESPONSE: Thank you; this has been updated in the introduction section on **page 5**, with reference to 2 additional studies (*Castro-Piñero et al. 2009. Criterion-related validity of field-based fitness tests in youth: A systematic review. Br J Sports Med;* and *Wind et al. 2010. Is grip strength a predictor for total muscle strength in healthy children, adolescents, and young adults? Eur J Pediatr.)*, as follows:

“Benefits of muscle may also be reflected in strength which can be measured directly for isolated arm muscles using handgrip tests, which correlate well (>0.7) with strength in other muscle groups such as hips and is thus a widely used proxy for overall muscular strength in larger scale studies (14-16).”

*6. Page 4, lines 1 and 2. This sentence is unclear. Why would you expect a sex difference here? Is this an example of HARKing?*

RESPONSE: Thank you; we agree that this needs more explanation. As mentioned above in response to Reviewer 2, our examination of sex differences here is motivated by previous evidence on sex differences in the associations of fat mass with these same atherogenic traits measured using targeted metabolomics; previous studies (now referenced) suggested more adverse patterns of association among males than females, with these sex differences in turn varying by life stage. These sex differences have not been previously examined for muscle strength or muscle mass, and our approach here is more agnostic and holistic such that we present results in full in supplementary files. We have expanded our rationale for this in the introductory section on **page 6** as follows:

“Growing evidence suggests that associations between body fat mass and atherogenic traits differ importantly by sex. Higher fat mass, whether measured using BMI, waist circumference, or DXA fat mass, appears to be more strongly positively associated among males with CVD-relevant traits including metabolomics-derived glucose, apolipoprotein-B lipids, and inflammatory glycoprotein acetyls (GlycA) (7, 28); these sex differences in turn vary by stage of body development with associations appearing more adverse among males in childhood, adolescence, and young adulthood, but similar or more adverse among females in middle adulthood (28). Such differences do not simply reflect differences in total fat volume since males tend to carry less total fat than females throughout life (28, 29); this instead suggests an important role of abdominal and ectopic fat storage, which is often higher among males (4, 29), in underpinning the effects of total fat (10). Males tend to have higher lean mass and stronger grip than females (14, 30), but whether important sex differences exist in the associations of muscle mass or strength with atherogenic traits is unknown. Sex differences in the potential benefits of muscle have also not been previously examined in relation to detailed atherogenic traits measured from targeted metabolomics (31). Muscle tissue is thought to be more modifiable after childhood (32) and thus greater benefits from a higher contractile capacity may be expected from adolescence onwards; but the common lack of repeated measures of body muscle at different life stages has prevented examination of the potential modifying role of growth and development in the associations of muscle change with atherogenic traits (33).”

**Requests from the editors**

*1. At this stage, we ask that you include a short, non-technical Author Summary of your research to make findings accessible to a wide audience that includes both scientists and non-scientists. The Author Summary should immediately follow the Abstract in your revised manuscript. This text is subject to editorial change and should be distinct from the scientific abstract.*

RESPONSE: Thank you; we have added a non-technical author summary after the abstract, on **pages 3-4** as follows:

“**Why was this study done?**

•	Higher body fat likely causes heart disease, but fat loss remains difficult to maintain. Evidence is less robust on whether gaining body muscle mass or strength would reduce risk of heart disease, and how the size of these potential benefits compares with the expected harms of fat gain. Whether changes in muscle and fat are more or less impactful during different stages of body development, or by sex, have not been previously examined. Linking these exposures to detailed blood measures of heart disease susceptibility from new metabolomics techniques could also help identify causal pathways,

•	Examining naturally occurring changes in lean mass, grip strength, and fat mass across early stages of life, when ageing-related chronic diseases are rare, should naturally reduce the potential for reverse causation and enable less biased estimates of effect of each body compartment on markers of heart health.

**What did the researchers do and find?**

•	We used data on ~3,000 young people from a British birth cohort study to examine repeated measures of body fat and lean mass taken from full body scanning performed during childhood, adolescence, and young adulthood, as well as repeated measures of handgrip strength from childhood and young adulthood, in relation to detailed factors taken from blood samples in young adulthood such as cholesterol which reflect one’s susceptibility to heart disease. This enabled us to compare how strongly different body compartments relate to heart health, and to pinpoint at what life stage body fat and muscle may most impact on health.

•	We found that gaining lean mass and grip strength were only weakly related to healthier levels of blood markers in young adulthood, particularly among males, with only lean mass gains occurring in adolescence appearing potentially beneficial. Gaining fat mass was more strongly and consistently related to poorer health in young adulthood, again particularly among males.

**What do these findings mean?**

•	These findings suggest that greater benefits to heart health may be expected from reducing body fat than from gaining body muscle. They further suggest that the regular use of muscle matters more than the volume or intentional building-up of muscle for avoiding heart disease.

•	Body muscle is still likely to benefit other functional aspects of health including mobility, and these should still be relayed to patients and the public.”

*2. Please clarify if the analytical approach reported here corresponded to one laid out in a prospective protocol or analysis plan? Please state this (either way) early in the Methods section.*

*a) If a prospective analysis plan (from your funding proposal, IRB or other ethics committee submission, study protocol, or other planning document written before analyzing the data) was used in designing the study, please include the relevant prospectively written document with your revised manuscript as a Supporting Information file to be published alongside your study, and cite it in the Methods section. A legend for this file should be included at the end of your manuscript.*

*b) If no such document exists, please make sure that the Methods section transparently describes when analyses were planned, and when/why any data-driven changes to analyses took place.*

*c) In either case, changes in the analysis-- including those made in response to peer review comments-- should be identified as such in the Methods section of the paper, with rationale.*

RESPONSE: A summary of our study’s aims, objectives, and analytical intentions was written prior to data handling as part of a data application submitted to ALSPAC in March 2019. This is now included as a Supporting Information file and described in the methods section on **page 6** as follows:

“Our study aims, objectives, and analytical intentions were summarised in March 2019 prior to data handling for the purposes of an ALSPAC data application (**Supporting Information 1**). This study was initially motivated by metabolomics work on type 2 diabetes susceptibility (41) and was expanded to investigate CVD susceptibility more broadly. Examinations of sex differences were motivated by more recent work on body fatness (30) and prior peer review recommendations.”

We have also noted 2 aspects of analyses which were changed after peer review (re-deriving relative grip strength based on body weight and cross-sectional analyses), on **page 9** as follows:

“Grip strength as a function of body weight, here termed ‘relative grip’, was calculated as maximum grip divided by body weight in kg. We scaled for weight rather than fat mass index or BMI when deriving relative grip following peer review; this was considered preferable because this: 1) accounts for overall body size rather than adiposity specifically; 2) reduces bias from an induced negative correlation between relative grip strength and adiposity; 3) enables consistent multivariable adjustment for fat mass index as a confounder across muscle measures; and 4) enables comparable estimates of effect with MR analyses given that genome-wide association studies have scaled for weight rather than BMI (43).”

And **page 11** as follows:

“Lastly, following peer review, we examined cross-sectional associations of lean mass indices, grip strength, and fat mass index with cardiometabolic traits, all measured at 25y, to compare the association profile of changes in exposures to that of current levels of exposure. These models were adjusted for the same covariates as in models with exposure change measured starting from 18y, but with smoking, alcohol, and lean/fat mass indices now measured at 25y.”

3. We'd suggest ensuring that the study is reported according to the STROBE guideline, and the completed STROBE checklist should be included as Supporting Information with the revised paper. Please add the following statement, or similar, to the Methods: "This study is reported as per the Strengthening the Reporting of Observational Studies in Epidemiology (STROBE) guideline (S1 Checklist)." The STROBE guideline can be found here: http://www.equator-network.org/reporting-guidelines/strobe/. When completing the checklist, please use section and paragraph numbers, rather than page numbers.

RESPONSE: Thank you; we have updated the STROBE checklist that was included with the original submission. This is now included as **Supporting Information 2** and mentioned in the methods section on **page 8** as follows:

“This study is reported as per the Strengthening the Reporting of Observational Studies in Epidemiology (STROBE) guideline (**Supporting Information 2**).”
