# Supplementary material for: Body muscle gain and markers of cardiovascular disease susceptibility in young adulthood: A cohort study
Source: PLoS Med. 2021 Sep 9;18(9):e1003751. doi: 10.1371/journal.pmed.1003751 (PMC8428664; doi:10.1371/journal.pmed.1003751)
Supplement: Attachment — Submitted filename: Response to Reviewers_R2.docx [file pmed.1003751.s046.docx]

**Response to Reviewers**

We thank the editors and reviewers for constructive comments on our manuscript, now titled **‘Body muscle gain and markers of cardiovascular disease susceptibility in young adulthood: A cohort study’**. These comments are copied below, followed by our responses.

**Reviewer 3**

*1. Looking through the list of covariates adjusted for, I notice that baseline values for the outcome of interest were not included. I think that it would be helpful to add this into the models. In particular it seems that the cross-sectional assocation between limb lean mass index and atherogenic traits, while weak, are directionally different from the assocations between changes in limb lean mass index and athergenic traits. I wonder if this lack of adjustment for baseline values in the atherogenic traits contributes?*

RESPONSE: We appreciate this suggestion but do not consider adjustment for baseline metabolic trait values to be appropriate here.

Our study aims to improve causal inference and covariate adjustments are strictly limited to factors which are suspected to be confounders, i.e., those which causally influence the outcome (metabolic traits at age 25 years) and the exposure (body fat/lean mass and grip strength across ages 10 to 25 years). Whilst baseline metabolic trait values may influence those same metabolic traits at 25 years, there is no strong evidence that baseline metabolic traits would influence our exposures. There is, however, strong evidence from prospective observational and Mendelian randomization studies (e.g. Würtz et al. PLoS Med: https://doi.org/10.1371/journal.pmed.1001765; Bull et al. BMC Med. 2020. https://doi.org/10.1186/s12916-020-01855-9) to support the forward direction of effect, at least for body fat mass: from fat mass to metabolic traits. Much less evidence exists on the potential effects of lean mass and grip strength on these metabolic traits (hence the present study), but prior evidence (current references 20-29) is still more supportive of this direction than the reverse.

Such baseline adjustments are also not consistently feasible, as these would require the same metabolic traits to be additionally measured at the same time as, or shortly before, each of our exposure measures. ALSPAC is one of the few cohorts with repeated metabolomics (available at ages 8, 15, 18, and 25 years), but these do not always align with the different measures of exposure change examined presently. For fat/lean mass change these include 10 to 25 years (overall), 10 to 13 years (childhood), 13 to 18 years (adolescence), and 18 to 25 years (young adulthood); whereas our exposure change measures for grip strength are from 12 to 25 years (overall only). We would not be able to apply baseline metabolic trait adjustments consistently across exposure time points, particularly for grip strength and adolescent fat/lean mass change (where the only available baseline metabolic measures are from ~4-5 years before exposure).

Properly examining potential biases induced from adjusting for baseline metabolic trait values would also require comparing results with versus without these adjustments, adding ~30,000 additional estimates to an already large volume of results (when considering different exposure-outcome pairs, adjustment stages, sex differences, sample selections): this would be very difficult to interpret and digest within a single paper.

*2. Values for grip strength appeared to decrease on average between age 12 and 25. This is very surprising, particularly in men - we would expect a 25 year old man to be considerably stronger than a 12 year old boy. Values for grip strength at age 25 are also vey low - less than half the level of that in UK Biobank in adults aged 40-70 years, who you would expect to be less strong than 25 year olds. Indeed, mean values are in the sarcopenic range. If there are errors in this measurement, this would call the entire analysis related to grip strength in this manuscript into question. An initial thought is that you might have inadvertently switched the values for 12 vs 25 years, but something else may be going on? Please could you recheck your data. It is not credible for grip strength amongst healthy 25 year olds to be this low.*

RESPONSE: We greatly appreciate this point which has led to several text corrections. The values previously shown for grip strength at age 25 years were indeed implausibly low for kg units and this was due to an error in the labelling of data received by the cohort study. The maximum grip strength variable at 25 years was initially labelled as having kg units, where this is actually measured in pounds per square inch (lb/in^2^) based on a different dynamometer than used in the clinic when aged 12 years. The clinic documentation for the age 25-year measurement also did not indicate that the device used was different from the previous occasion. These clinic data are relatively new, and our study is the first to publish on this new grip strength measure. These labelling errors are being corrected by the study team for future data users.

This does not have substantial implications for the present study: both types of dynamometer (the Jamar hydraulic type used at age 12 years and the Baseline Pneumatic Squeeze bulb type used at age 25 years) are commonly used to assess grip strength and are well correlated in past studies (r > 0.8; new references added in text: Li et al. Man Ther. 2010. https://doi.org/10.1016/j.math.2010.06.010; and Bobos et al. Arch Phys Med Rehab. 2020. https://doi.org/10.1016/j.apmr.2019.10.183). Importantly, our main analyses are based on standardised (z-score) units of exposures, including for change scores which are based on standardised measure at age 25 years minus standardised measure at age 12 years), which should enable comparison of effect sizes across exposures with different measurement units. This does impact the histogram of grip strength change provided in **Supplementary Figure 10**, however, and we have updated this to be based on SD units of change and clarified the original units in **Table 1**. We have updated our description of grip strength measures in the methods section on **page 8-9** as follows:

*“Participants underwent handgrip strength testing on two occasions: when aged approximately 12 years using a Jamar hydraulic dynamometer and when aged approximately 25 years using a Baseline Pneumatic Squeeze bulb dynamometer (the latter device records grip more dynamically with units scaled to hand size; previous studies of younger and older adults suggest that such devices are highly correlated with Jamar hydraulic devices (r > 0.8) and similarly detect sex differences in grip strength (43, 44)). On each occasion, participants sat in a chair with arms and back supported and were asked to rest their forearms on the arms of the chair with their wrist just over the end of the chair arm (thumb facing upwards with wrist in a neutral position). Participants were asked to squeeze the device as tightly and for as long as possible, three times in succession using their dominant (writing) hand, to record maximum isometric strength (in kilograms (kg) of force at the 12-year occasion and in pounds per square inch (lb/in^2^) of force at the 25-year occasion).”*

And on **page 10** as follows:

*“Exposures and outcomes were analysed in standardised z-score units to allow comparability of effect sizes given dissimilar variances between traits and across occasions (this also improved comparability between handgrip strength measures given different units across occasions).”*

*3. Table 1, please could you also report data for males and females separately. This is important for values such as grip strength and fat and lean mass indexes which will differ substantially according to sex.*

RESPONSE: This is a useful addition which also complements the sex-specific histograms provided in supplementary files. We have added sex-stratified descriptive results in **Table 1** and **Supplementary Table 1**.

*4. I am not sure that I fully agree with this statement in the "Why was this study done?" section: "Examining naturally occurring changes in lean mass, grip strength, and fat mass across early stages of life, when ageing-related chronic diseases are rare, should naturally reduce the potential for reverse causation and enable less biased estimates of effect of each body compartment on markers of heart health.". I would accept this argument if you were comparing changes from say 18 to 30, but not so much for changes from 12 to 25, when developmental changes are likely to play a substantial confounding role that you cannot fully adjust for. Similarly, I am not sure I fully agree with the earlier comment "This enabled us to compare how strongly different body compartments relate to heart health, and to pinpoint at what life stage body fat and muscle may most impact on health." You did not look at several life stages here - just adolescence - so both these statements need to be tempered.*

RESPONSE: We consider the first statement about younger ages reducing the potential for bias to be valid but agree this could be more specific and have changed the term “reverse causation” to “confounding by subclinical disease” on **page 3**. Potential confounding by early life developmental changes is unrelated to the point about confounding from subclinical disease, i.e., a major problem when analysing samples of older adults where lower/weaker muscle may be a result, rather than a cause, of metabolic trait levels. Developmental changes, if stemming from puberty, cannot be a confounder of several exposures measured here as these exposures are measured before the onset of puberty: pubertal growth may instead be a mediator (which should not be adjusted for). Additionally, apparent effects of developmental changes may themselves be confounded. In a previous study using prospective observational, negative control, and Mendelian randomization methods in the same ALSPAC cohort (Bell et al. PLoS Med: https://doi.org/10.1371/journal.pmed.1002641), we found strong evidence that the apparent effects of puberty timing on levels of body fat mass, lean mass, and these same metabolic traits measured at age 18 years are likely confounded by fat mass in childhood, i.e., there is unlikely to be an influence of puberty timing itself on these metabolic traits.

Regarding the second statement about life stages, we agree this can be tempered and we have clarified that we are referring to stages of early life, before adulthood, on **page 4**. The full scope of data used spans age 10 to 25 years, which includes pre- and post-adolescence based on objective height-based measures of pubertal onset. We also include a separate set of analyses of age 18 to 25 years, which we consider to be more specific to young adulthood given that pubertal changes have likely ended by this point.

**Requests from editors**

*1. Please remove the word "prospective" from the title, and adapt the study descriptor to "A cohort study".*

RESPONSE: This has been updated.

*2. Please quote study dates in the abstract.*

RESPONSE: There are 5 different measurement occasions involved in these analyses of ALSPAC birth cohort study data; adding dates at each mention of an age makes the abstract difficult to read and so we have added the dates of main study recruitment and the range of dates of clinics used after their first mention in the methods section as follows:

*“Data were from 3,227 offspring of the Avon Longitudinal Study of Parents and Children (39% male; recruited in 1991-92). Limb lean and total fat mass indices (kg/m^2^) were measured using dual-energy X-ray absorptiometry scans performed when aged 10, 13, 18, and 25 years (across clinics occurring in 2001-03 to 2015-17).”*

*3. Please restructure the author summary to consist of three subsections, each of 3-4 short points (1-2 short sentences each).*

RESPONSE: The author summary is currently in the format requested (3 subsections, each with 3-4 short points, each point involving 1-2 sentences); we have shortened several points where feasible for brevity.

*4. Please specify "Written informed consent" in the Methods section, assuming this is correct.*

RESPONSE: This has been updated on **page 7** of the methods section.

*5. Please spell out "years" throughout the text.*

RESPONSE: This has been updated throughout the main text. We have kept this abbreviation in tables and figures for brevity.

*6. Throughout the article, please adapt the reference call-outs to the following style: "... and glucose [6,7]." (noting the absence of spaces within the square brackets).*

RESPONSE: This has been updated.

*7. Please remove the information on funding, competing interests and data access from the end of the main text. In the event of publication, this will appear in the article metadata, via entries in the submission form.*

RESPONSE: These sections have been removed on **page 20**.

*8. Please make that "PLoS ONE" in the reference list.*

RESPONSE: This has been updated.

*9. Please add "[preprint]" to reference 30 and any other preprints cited.*

RESPONSE: This has been updated.

*10. Noting references 41 & 48, please ensure that all citations have full access information.*

RESPONSE: These have been updated.

*11. Please rename the STROBE checklist "S2_STROBE_Checkist", or similar, and refer to it as such in the Methods section.*

RESPONSE: We have renamed this file as ‘S2 STROBE Checklist’ in the methods text and file name. The previously named ‘Supporting Information 1’ was also renamed as ‘S1 Study Plan’ to match this format.
