# Supplementary material for: Body muscle gain and markers of cardiovascular disease susceptibility in young adulthood: A cohort study
Source: PLoS Med. 2021 Sep 9;18(9):e1003751. doi: 10.1371/journal.pmed.1003751 (PMC8428664; doi:10.1371/journal.pmed.1003751)
Supplement: Attachment — Submitted filename: Response to Reviewers_R3.docx [file pmed.1003751.s047.docx]

**Response to Reviewers: ‘Body muscle gain and markers of cardiovascular disease susceptibility in young adulthood: A cohort study’**

We thank the editors and reviewers for their constructive comments throughout the peer review process. The remaining editorial comments and responses to these are copied below.

*1. Please add a few words to your data statement (submission form) to explain the reasons that data cannot be made openly available, e.g., "Owing to constraints of the study's ethics approval, individual-level ALSPAC data are available upon application only ...".*

RESPONSE: This has been updated via the online submission form, after consulting the ALSPAC data access policy, as follows:

*The informed consent obtained from ALSPAC participants does not allow the data to be made freely available through any third party maintained public repository. However, data used for this submission can be made available on request to the ALSPAC Executive. The ALSPAC data management plan describes in detail the policy regarding data sharing, which is through a system of managed open access. Full instructions for applying for data access can be found here: http://www.bristol.ac.uk/alspac/researchers/access.*

*2. Please adapt the "Conclusions" subsection of your abstract to begin: "In this study, we found that ... was weakly associated ..." or similar.*

RESPONSE: The first sentence of the conclusion section of the abstract has been updated on **page 3** (and on the online abstract section) as follows:

*In this study, we found that muscle strengthening, as indicated by grip strength gain, was weakly associated with lower atherogenic trait levels in young adulthood, at a smaller magnitude than unfavourable associations of fat mass gain.*

*3. Please avoid the long bulleted point in the second subsection of your Author summary. This could be split into two points, perhaps.*

RESPONSE: The first point under the ‘What did the researchers do and find?’ section of the author summary has been split into 2 shorter sections on **page 3-4** as follows:

*We used data on ~3,000 young people from a British birth cohort study to examine repeated measures of body fat and lean mass taken from body scanning performed during childhood, adolescence, and young adulthood, as well as repeated measures of handgrip strength from childhood and young adulthood.*

*We examined associations between these exposures and detailed factors taken from blood samples in young adulthood including apolipoprotein-B-related cholesterol which reflects susceptibility to heart disease. This enabled us to compare how strongly different body compartments relate to heart health and to pinpoint at what stage of early life (before adulthood) each may be most impactful.*
